# Comparative Study of Zeolite Overlayers on ZnO Gas Sensors: Effects of Framework Structure on Sensing Performance

**DOI:** 10.3390/s26206364

**Published:** 2026-10-09

**Authors:** Valerie J. J. Ries, Paula Tarttelin Hernández, Ivan P. Parkin

**Affiliations:** 1 Materials Chemistry Centre, Department of Chemistry, University College London, London WC1H 0AJ, UK; valerie.j.j.ries@gmail.com (V.J.J.R.); i.p.parkin@ucl.ac.uk (I.P.P.); 2 Centre for Forensic Science, MAPS School, University of Technology Sydney, Ultimo 2007, Australia

**Keywords:** gas sensors, metal oxide semiconductors, zeolite modification

## Abstract

Zeolite incorporation into metal oxide semiconductor (MOS) gas sensors offers a promising way of improving sensor sensitivity, vapour discrimination, and operating performance. However, zeolite overlayers may also affect vapour diffusion, response kinetics, and responsiveness to target vapours. In this study, screen-printed ZnO thick-film sensors were modified with four structurally different zeolites (H-Y, H-Beta, Na-A, and H-ZSM-5) to investigate the effect of framework properties on sensing behaviour. The resulting sensor array was evaluated towards nitrogen dioxide (NO_2_), ethanol, acetone, propane, and carbon monoxide. Na-A modification (sensor ZnO-LTA) produced the greatest enhancement towards NO_2_, increasing the response to 900 ppb NO_2_ by 2.8-fold (R_max_ = 28) at 350 °C and reducing response and recovery times by 20–30 s. ZnO-LTA exhibited the highest response towards 60 ppm ethanol, reaching R_0_/R = 62.3 at 350 °C, while ZnO-MFI showed the highest acetone response, achieving R_0_/R = 177.4 at 350 °C. Structural characterisation by XRD, Raman spectroscopy, SEM, and EDX confirmed preservation of both the ZnO phase and zeolite framework structures following sensor fabrication. This manuscript provides the first systematic comparison of four zeolite frameworks applied as screen-printed overlayers on ZnO and shows how zeolite framework characteristics influence sensor behaviour, operating temperature, and response dynamics. The findings highlight the potential of zeolite-modified ZnO sensors for vapour discrimination in MOS-based sensor arrays.

## 1. Introduction

Integrating metal oxide semiconductor (MOS) gas sensors into an electronic nose platform necessitates devices that are small, portable, robust, and cost-effective [1]. Fundamentally, they must be sensitive to low vapour concentrations in the ppm-ppb range and exhibit selectivity towards target vapours without interference in mixed-gas environments [2]. MOS sensors are attractive in this context because they are highly sensitive, inexpensive to fabricate, offer fast response times and potential for miniaturisation, and are already widely used as portable systems in environmental monitoring applications [3,4,5,6]. They have been applied in fields ranging from pollution monitoring [7,8], to medical diagnostics [9], to the detection of food spoilage biomarkers [10], hazardous gas detection [11], and security applications involving explosive and flammable gases [12]. This type of system, however, often requires the continuous application of a voltage or current to obtain a measurable signal [13]. Furthermore, elevated temperatures are generally needed for thermal activation—this facilitates reactions between the gases of interest and the sensing material which, in turn, increases the power consumption of the sensors and limits opportunities for miniaturisation [13].

Zinc Oxide (ZnO) is among the most studied MOS sensing materials, first explored as a gas sensor by Seiyama et al. (1963) [14]. It is a wide bandgap (~3.7 eV) n-type II-VI semiconductor and has a high exciton binding energy at room temperature of 60 meV [15,16]. The sensor’s response is dependent, among other things, on the surface reactions between the gases of interest and the sensing material, which are strongly influenced by both the morphology and defects in the sensing material’s crystalline structure [17]. Upon exposure to a gas, the baseline resistance of the sensor changes in a way that can be measured [17]. Alongside SnO_2_ and In_2_O_3_, ZnO is valued for its high sensitivity to both reducing and oxidising gases, chemical and thermal stability, high electron mobility, low toxicity, and low production cost [16,18,19,20,21]. Yet unmodified ZnO, like many other MOS materials, suffers from poor intrinsic selectivity to vapours of interest, limiting its viability for deployment in real-world settings. In addition, ZnO requires high operating temperatures, which may raise user safety concerns, as well as adversely affect the sensor’s stability and signal drift [13,16]. Consequently, considerable research has focused on modifying MOS materials to reduce operating temperatures and improve sensing performance. Recent advancements in this field include: introducing morphological changes and ZnO/SnO_2_ heterojunction engineering for n-butanol detection [22]; 3D ZnO/SnO_2_ nanorod heterostructures grown on silica micropillar arrays for enhanced ethanol detection [23]; electrospun ZnO-based nanocomposites for biomarker and reducing gas detection [24,25]; the creation of high-density active sites in sol–gel-derived ZnO for carbon monoxide (CO) and carbon dioxide (CO_2_) sensing [17]; catalytic additives such as Pd nanoparticles for enhanced ethanol detection [15]; doping [26]; surface functionalisation strategies such as ZIF-8 derived ZnO nanosheets-functionalised mesoporous In_2_O_3_ for formaldehyde detection [27]; and the incorporation of selective filters or admixtures through zeolite incorporation [28]. Further examples of sensing material modifications aimed at enhancing sensor selectivity capabilities can be found in [29,30,31,32].

Among the available functional modifiers, zeolites represent one of the most versatile classes of materials for strategic tuning of MOS sensor performance. They are crystalline aluminosilicates composed of interconnected SiO_4_ and AlO_4_ tetrahedra that generate a three-dimensional network of microporous channels and cavities of molecular dimensions [33]. A negative charge is introduced into the zeolite framework when silicon ions are replaced by aluminium ions [34]. This negative charge is compensated by ion-exchangeable extra-framework cations that are electrostatically bound to the framework but remain mobile within the zeolite pores and channels [34]. These cations have a direct effect on sensing behaviour; they can affect gas diffusion and adsorption, and can also act as acidic sites that favour catalytic reactions in the sensor system [34,35]. When sufficient thermal energy is supplied, the extra-framework cations can move between preferential sites within the framework. Prior studies suggest that the introduction of a target gas affects this cation mobility, resulting in measurable changes in sensor response [35]. The Si/Al ratio of zeolite framework determines the number of cations and the hydrophobic/hydrophilic character of the zeolite. These properties have been exploited to promote selective adsorption based on the polar character of target molecules [35]. Another useful property of zeolites is that their pore openings can act as molecular sieves, allowing size- and shape-selective diffusion of gas molecules [35]. In gas-sensing applications, when zeolites are applied as overlayers, they have been shown to enhance discrimination between chemically similar vapours. Furthermore, their tuneable acidity, ion-exchange properties, internal microporosity and high surface area lead to increased adsorption sites, providing additional catalytic and adsorption-based mechanisms able to modify the gas composition reaching the sensing layer [30,35,36,37]. Nevertheless, zeolite integration can also introduce challenges such as slower response and recovery times or reduced responsiveness to target gases when used in single sensor systems [1,30]. Consequently, single and unmodified sensor systems remain insufficient for gas detection in complex environments, and sensor arrays—where each sensor is functionally differentiated—are required to achieve effective vapour discrimination [38].

Although zeolite–MOS combinations have been explored in several systems, including SnO_2_, WO_3_, In_2_O_3_, and Cr_2_O_3_ with zeolites such as H-ZSM-5, TS-1, and Na-A [4,12,29,39,40], these studies have not examined how different zeolite framework structures influence ZnO specifically. Moreover, existing work typically employs hydrothermal growth or spin-coating rather than more scalable screen-printing fabrication approaches [41,42]. As a result, the effects of pore architecture, Si/Al ratio, and zeolite hydrophilicity/hydrophobicity on ZnO sensor behaviour remain largely unexplored. To the best of our knowledge, this study provides the first investigation of screen-printed thick-film ZnO sensors modified with four structurally distinct zeolites, namely H-ZSM-5, Na-A, H-Y, and H-Beta, when applied as overlayers on the ZnO sensing material. The study has focused on the detection of vapours with environmental, safety, medical, security, and forensic relevance. Propane detection is important for leak detection and explosion prevention in domestic and industrial settings and has also been reported as a recreationally inhaled substance [43,44]. Ethanol is widely used in food, chemical, biomedical and transport industries and is a common interfering vapour in gas-sensing applications [15]. Acetone is of interest as a breath biomarker for diabetes and is also frequently used as a solvent in clandestine drug manufacture [45,46]. Carbon monoxide is a toxic atmospheric pollutant and remains an important gas for safety monitoring [17]. NO_2_ exposure has been associated with adverse health effects and nitro-containing compounds have also been investigated as markers of explosives and illicit drug materials [12].

This work provides novel insights into the sensing performance of thick-film ZnO-based sensors with four structurally distinct zeolite overlayers via screen-printing, a scalable sensor fabrication approach. It also details the observed relationships between zeolite framework characteristics and sensing behaviour through comparisons of sensor response magnitudes and patterns, operating temperature behaviour, and response and recovery dynamics during exposure to nitrogen dioxide, ethanol, carbon monoxide, acetone, and propane vapours.

## 2. Materials and Methods

### 2.1. Sensing Materials

Commercial ZnO was supplied by Sigma-Aldrich (Dorset, UK). Zeolite materials were supplied by the PG Corporation and Zeolyst International, Conshohocken, PA, USA.

### 2.2. Sensor Fabrication

**Figure 1:**
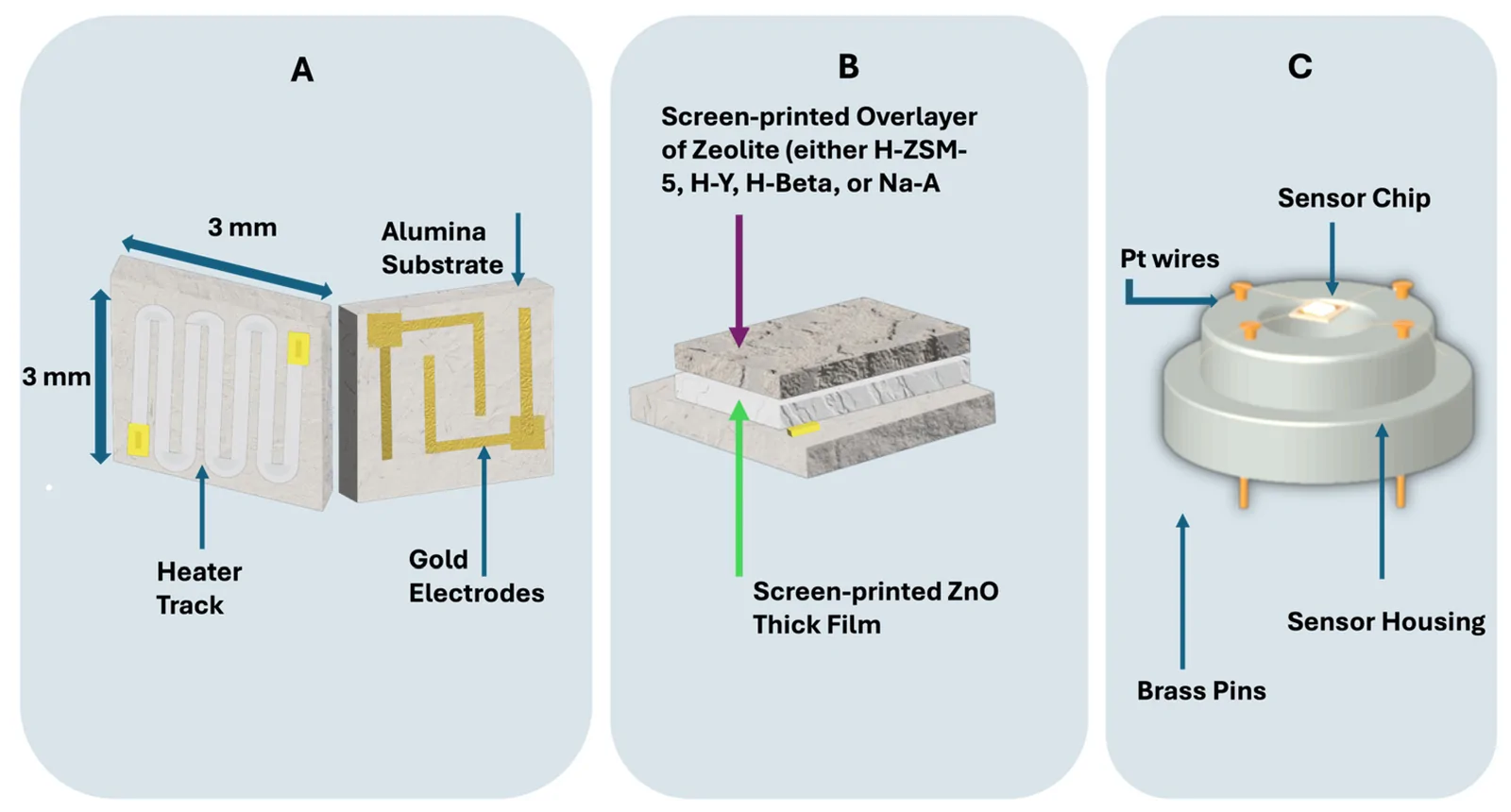
(**A**) Sensor chip characteristics. (**B**) Alumina substrate with screen-printed ZnO and zeolite overlayers printed on top of it. (**C**) Sensor housing showing the spot-welded sensor onto the housing’s pins.

**Figure 2:**
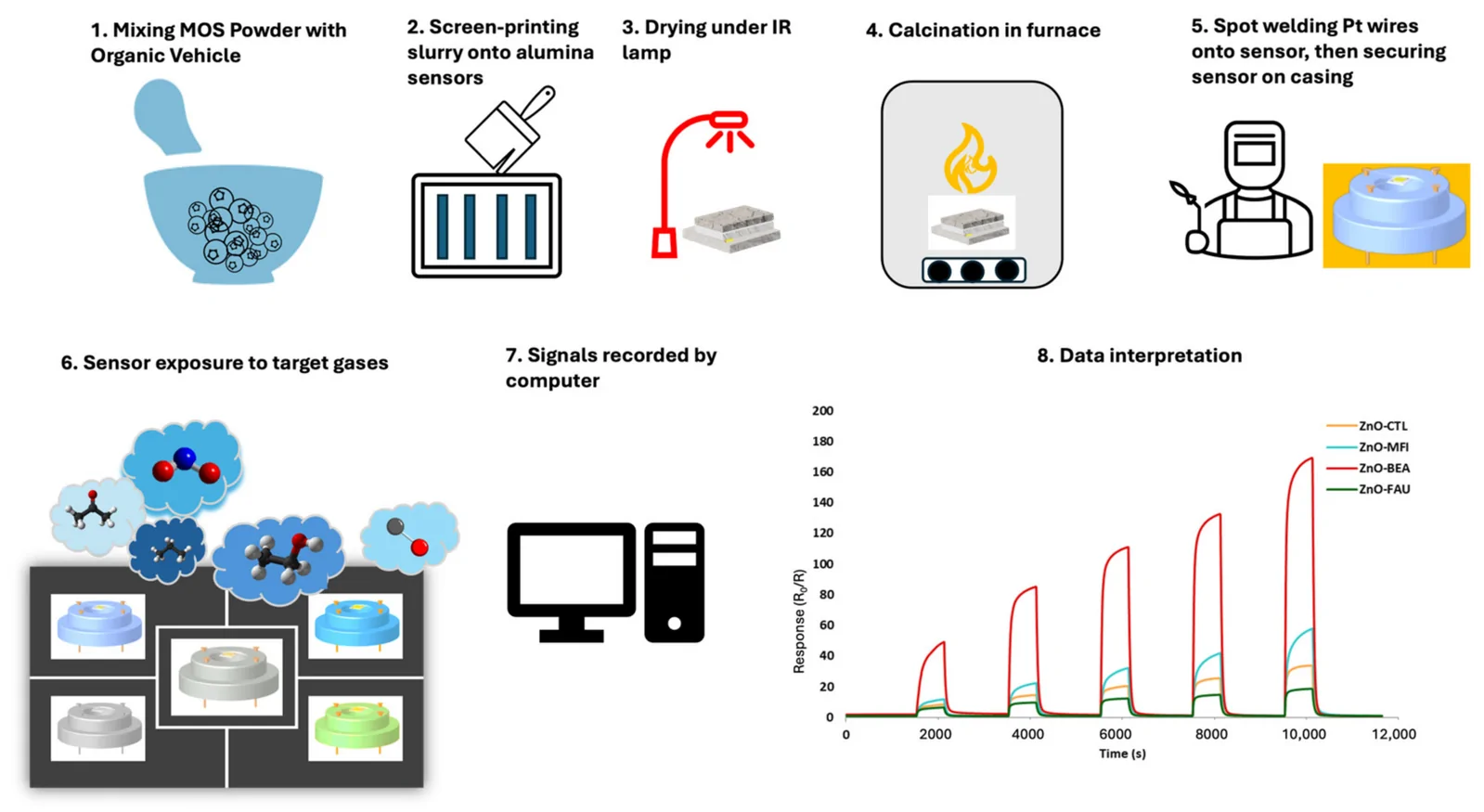
Thick-film MOS gas sensor fabrication and analysis process.

The fabrication procedure involves printing a sensing material ink onto a sensor chip, which then needs to be attached and housed in a system that sits in the gas-sensing rig (Figure 1). An ink suitable for screen-printing was produced by mixing the ZnO powder with an organic vehicle (ESL vehicle 400, Agnet, Ltd., London, UK) using a pestle and mortar for 30 min (Figure 2). The same procedure was used to create the zeolite-based ink as described in prior literature [12,36].

A DEK 1202 screen printer (DEK Printing Machines, Ltd. Weymouth, UK) was employed in combination with an MCI 3 × 3 precision screen with a patterned mesh that enabled the ink to be deposited between the two gold electrode pads and onto individual sensors on the strip (Figure 2). Pressure was inflicted with a squeegee, set to automatically move and transfer the ink through the mesh, covering the full length of the strip. After each deposition, the ink was allowed to dry under an IR lamp. For the single sensor material fabrication, five layers of the ZnO material were printed on the interdigitated gold electrodes. The incorporation of zeolite layers on top of the base material is straightforward, simply requiring layers of zeolite to be deposited on top of the sensing material using the same method. Following completion of the printing process, the sensors were sintered in a furnace (Elite Thermal Systems Ltd., Market Harborough, UK) at 600 °C for an hour, increasing the temperature at a rate of 25 °C/min. The sensor strip was then broken down to individual chips and a sensor with a specific location on the strip would be selected for testing to minimise any potential variability caused during the fabrication process.

A MacGregor DC601 spot welder (MacGregor Welding Systems, Mildenhall, UK) was used to connect the platinum wires to the electrodes on the sensor, i.e., the two gold electrode pads on the obverse of the sensor and the two platinum ones on the reverse of it. Then, the sensor was connected to the housing by spot welding the platinum wire to each of the four protruding terminals, as shown in Figure 1 and Figure 2.

Five sensors were prepared, as shown in Table 1. A control sensor was produced with commercially sourced ZnO. Modified sensors were made by screen-printing H-ZSM-5, Na-A, H-Beta, and H-Y zeolites on top of the ZnO material (Figure 1B). While the film thickness was not measured in this study, previous studies by the same group have assessed it to be *ca*. 130 µm for the sensing material and *ca*. 28 µm for the three layers of zeolite when measured with a Hitachi S-3400N microscope (Hitachi High-Tech Corporation, Tokyo, Japan) operated at 5 kV and using a working distance of approximately 16 mm [47].

### 2.3. Sensor Characterisation Techniques

Sensors were characterised using powder X-ray diffraction (XRD), Raman spectroscopy, Scanning Electron Microscopy (SEM) and Energy Dispersive X-ray Spectroscopy (EDX).

XRD was performed on a Bruker D8 General Area Detector Diffraction System (GADDS) diffractometer (Bruker Corporation, Billerica, MA, USA) using CuKα1 radiation (λ = 1.5406 nm). The X-ray source operated at 30 W with a Vantec 500 detector and a 1 mm collimator. X-ray patterns were obtained over a 2θ range of 7° to 65° and a time step of 100 s/step × 3 steps.

Raman spectroscopy was used to obtain structural information and provide a unique fingerprint for the materials analysed. Raman spectra were obtained on a Renishaw inViaTM microscope (Renishaw plc, Gloucestershire, UK) using an excitation laser of 514 nm.

Scanning electron images were captured using a JEOL JSM- 6301F microscope (JEOL Ltd., Tokyo, Japan). A 5 keV probe voltage was used in the secondary electron imaging mode. Samples were carbon-coated before analysis with an Edwards S105B sputter-coater (Edwards High Vacuum/Edwards Ltd., West Sussex, UK) and images were captured with Semafore. A 20 keV probe voltage coupled to an Oxford Instrument INCA X-sight system (Oxford Instruments, Abingdon, UK) was used. As the samples were coated using carbon prior to doing the analysis, carbon was excluded from the atomic percentage calculations. The Oxford Instrument INCA X-sight system coupled to SEM was used to calculate the atomic percentage of the sensors.

Brunauer–Emmett–Teller (BET) was conducted on the powdered samples to determine the surface area. This was achieved using a micromeritics ASAP 2420 analyser (Micromeritics Instrument Corporation, Norcross, GA, USA). Degassing was performed at 150 °C under a flow of nitrogen gas for over 12 h. The material was weighed afterwards, and the porosity and surface area were determined using a Micromeritics TriStar II PLUS (Micromeritics Instrument Corporation, Norcross, GA, USA). BET precision ranges have been reported between 0.1 and 3.96% and measurement bias is generally within ±5% of the certified surface area of materials [48].

### 2.4. Gas-Sensing Protocol

**Figure 3:**
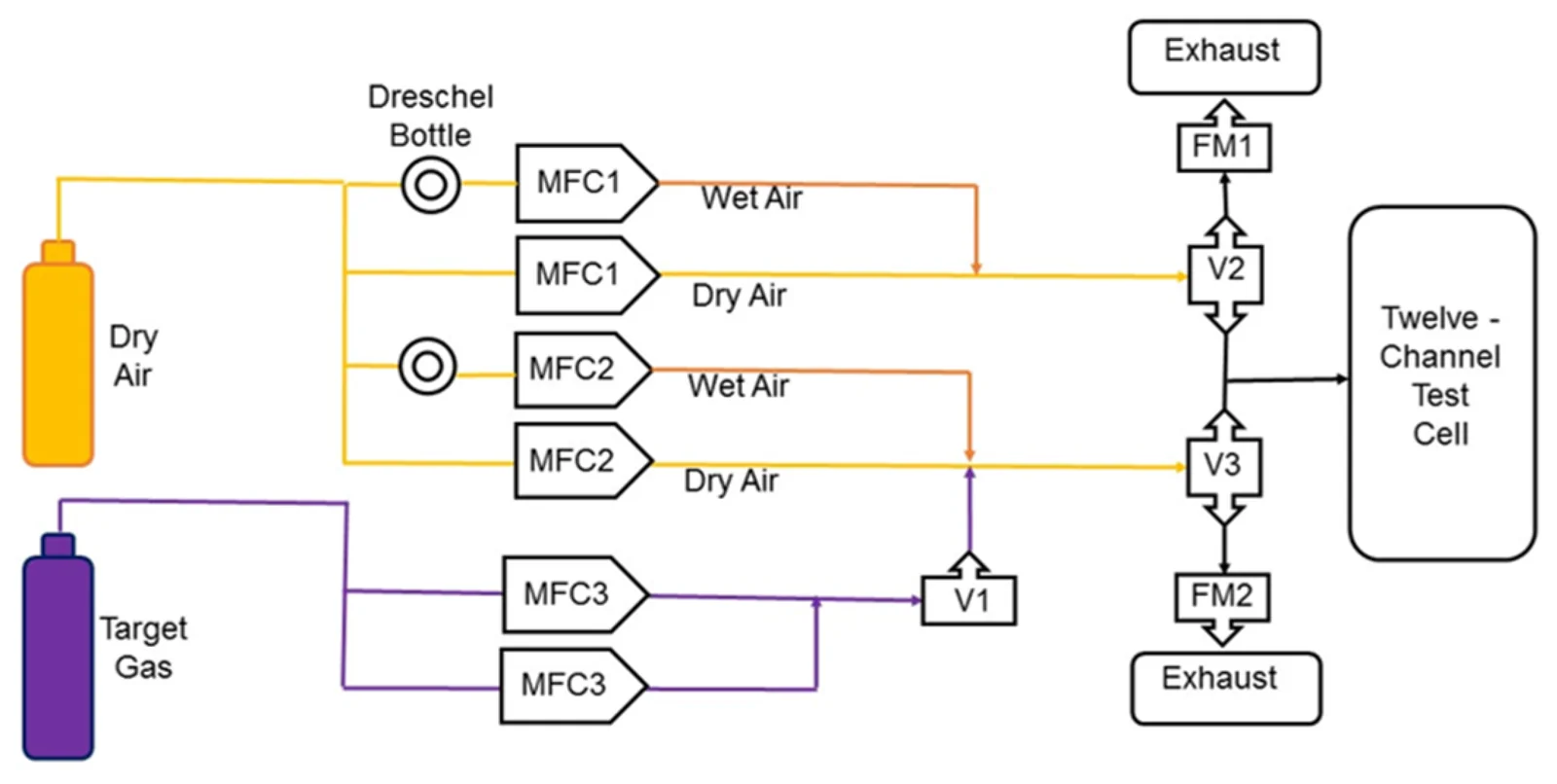
Schematic of UCL’s custom-built gas-sensing rig consisting of a test cell, mass flow controllers (MFC), flow metres (FM), solenoid valves (V) and Dreschel bottles, which were not operational in this study. Figure adapted from [49,50].

Gas exposure was performed on a custom-built rig, consisting of a twelve-channel chamber where sensors were simultaneously exposed to a flow of air and/or gas (Figure 3). Before gas exposure, sensors were exposed to dry air to establish their baseline resistance in air. Aera FC-7700C mass flow controllers (MFCs) (Aera Japan Ltd., Tokyo, Japan) were used, which could deliver an airflow of up to 1 L/min to the gas-sensing chamber [49]. The operation of the MFCs was managed through the rig’s computer programme Pascal, which enabled the desired flow of air/gas to be delivered into the gas-sensing chamber. Platon flow metres were utilised to ensure correct flow into the chamber. Bürkert 3/2 solenoid valves were used to control the sections of the system one wished to use to supply air and/or gas into the sensing chamber [49].

The sensor chamber could hold up to 12 sensors at any one time. The temperature of each sensor was kept constant during measurements and adjusted as needed via a Wheatstone bridge circuit [49]. The driving voltage required to heat the sensor to a certain temperature was calculated using the resistance curve of platinum and the room temperature resistance of the sensor’s platinum heater track, from four-point resistance measurements [49,50].

The sensor resistance was measured using a potential divider circuit and amplifier. A constant probe voltage of 100 mV was supplied by an inverting operational amplifier [49]. The sensor’s resistance was measured when exposed to an airflow of dry air (R_0_), and was compared to the exposure in a mixture of air and gas (R). For an n-type semiconducting material, gas responses to an oxidising gas are expressed as R/R_0_, whereas to a reducing gas they are expressed as R_0_/R. The response time (T_res_) is the time taken by the sensor to reach 90% of the maximum response once the gas is introduced into the system. The recovery time (T_rec_) is the time for the response to fall from the maximum response value to within 10% of the baseline upon gas removal and subsequent exposure to just air.

Various concentrations were tested from cylinder concentrations of nitrogen dioxide (1 ppm), ethanol (100 ppm), carbon monoxide (1000 ppm), acetone (100 ppm), and propane (100 ppm). NO_2_ concentrations are reported in ppb because the certified NO_2_ cylinder was diluted to sub-ppm levels to understand the sensors’ capabilities in approaching environmental and medical concentration ranges. All other analytes were supplied in ppm-level cylinders and are thus reported in ppm. Sensors were tested in the 300–500 °C temperature range, enabling determination of optimal operating temperatures. Once this was determined, sensors were exposed to various pulses of progressively increasing gas concentrations.

## 3. Results and Discussion

### 3.1. Materials Characterisation

The following sections describe the structural characterisation and gas-sensing evaluation of the sensor array created in this study.

The structural integrity, morphology, and elemental composition of the screen-printed ZnO and zeolite-modified sensors were examined using XRD, Raman spectroscopy, SEM and EDX to confirm successful fabrication and to understand how the zeolite overlayers might influence the ZnO sensing material.

#### 3.1.1. X-Ray Diffraction (XRD)

**Figure 4:**
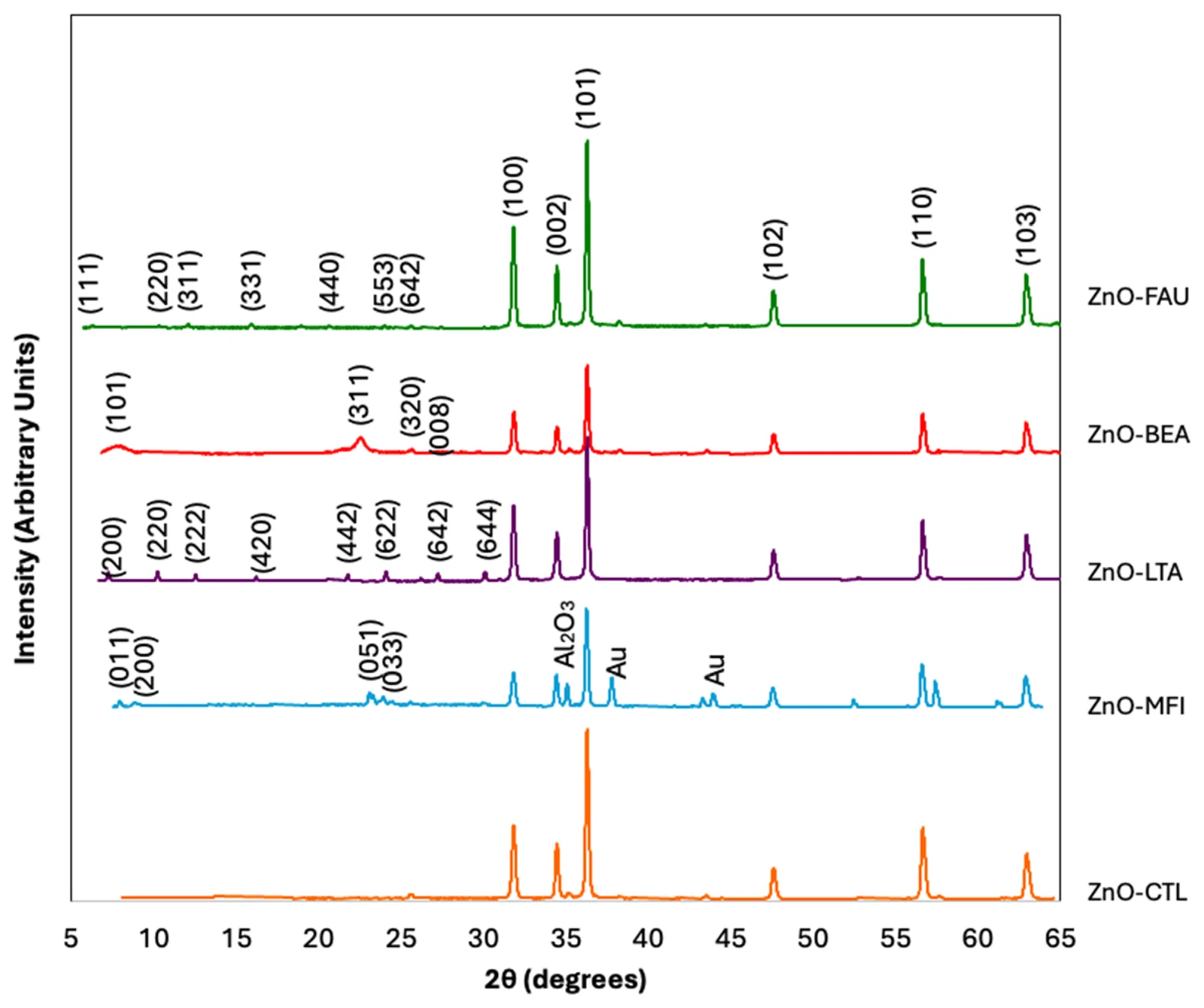
X-ray diffraction patterns for ZnO and zeolite overlayers collected over a 2θ range of 7–65°.

XRD patterns collected over 2θ = 7–65° confirmed that all sensors retained the characteristic diffraction peaks of hexagonal wurtzite ZnO, with major reflections at 2θ values: 31.8°, 34.4°, 36.3°, 47.5°, 56.6° and 62.9° (Figure 4). The XRD peaks obtained were indexed to the hexagonal wurtzite structure and ZnO values reported in the literature: (100), (002), (101), (102), (110) and (103) planes [51]. The absence of peak shifting indicates that the thermal treatment used during sensor fabrication did not induce distortion to the crystalline structure of the sensors. The zeolite-modified sensors displayed additional low-intensity peaks corresponding to the respective zeolite frameworks. Lower intensities relative to ZnO are expected, as zeolite crystals are smaller and were present only in the uppermost layers of the sensor.

More specifically, zeolite H-Y displayed a FAU structure and displayed 2θ peaks at values: 6.0°, 10.0°, 12.0°, 15.8°, 23.9° and 25.4°, in line with literature [52] and indexed as (111), (220), (311), (331), (440), (553) and (642) planes, respectively. Zeolite H-Beta had a broad peak at 2θ = 7.5° and three additional peaks at 2θ = 22.4°, 25.5° and 26.9°, confirming the BEA structure [53]. These peaks corresponded to the (101), (311), (320) and (008) planes. Characteristic peaks of the LTA framework of Na-A zeolite in the 2θ range of 5–30° were present giving indexed peaks for the (200), (220), (222), (420), (442), (622), (642) and (644) planes [54]. X-ray diffraction peaks and corresponding planes for H-ZSM-5 were (011), (200), (051) and (033) [55]. XRD results confirm that zeolites retained their crystalline structures after the sensor fabrication process. No secondary phases formed between the ZnO and zeolite materials and the zeolite overlayers did not affect the crystal lattice of ZnO.

To further evaluate structural changes induced by the different zeolite overlayers, crystallite sizes were estimated using Scherrer’s formula:

(1) $$D=\frac{k\lambda }{\beta cos\theta }$$

where *D* is the average crystal size, with all angles in radians. For spherical crystallites, generally, *k* is a constant ~0.9. λ corresponds to the X-ray wavelength (1.5406 Å), and β is the full width at half maximum (FWHM) of the peaks of the ZnO-based films. While the results show that the crystallite sizes may become a little bigger with zeolite incorporation, the difference is likely to be insignificant within the precision of the Scherrer estimate (Table 2).

The calculated values of the crystallite sizes were 32.2 nm (ZnO-CTL), 34.4 nm (H-ZSM-5), 32.2 nm (H-Beta), 35.3 nm (H-Y), and 46.9 nm (Na-A). These values indicate that zeolite incorporation does not disrupt the ZnO crystal structure; no significant peak shifts or phase impurities were observed. However, some variation in crystallite size was observed, particularly for LTA, which displayed the largest average crystallite size among sensor materials. The zeolite-modified sensors displayed different response patterns as detailed in forthcoming sections.

The porosity of the thick films could not be measured directly on the alumina substrates and BET was performed on the powdered samples, as illustrated in the ‘Zeolite Incorporation Effects’ section below. This is because the screen-printed films are adhered to the alumina substrates and cannot be removed without damaging the printed layers. Instead, porosity-related effects were inferred from the SEM microstructure and from intrinsic pore characteristics of the zeolite.

#### 3.1.2. Raman Spectroscopy

**Figure 5:**
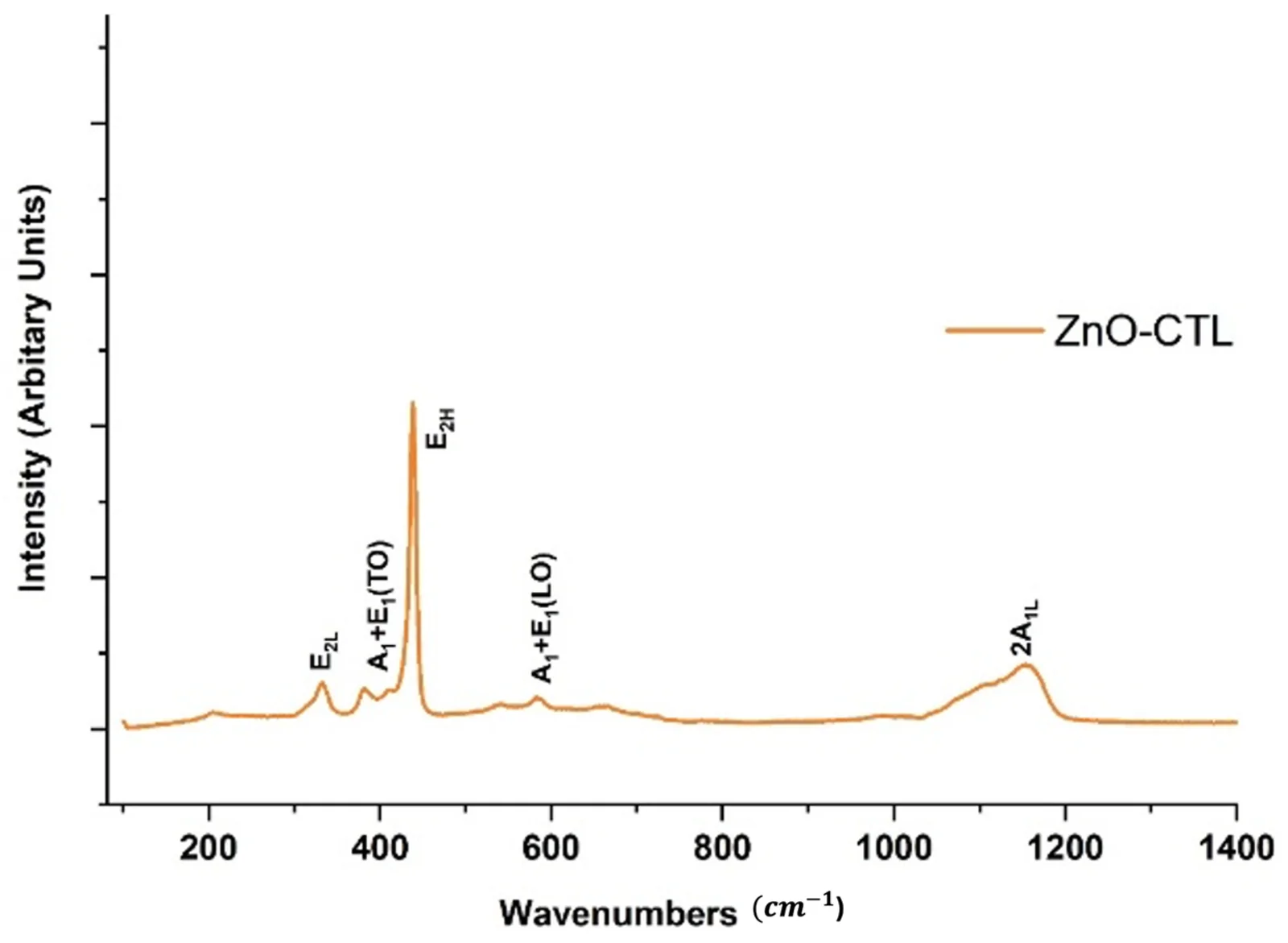
Raman spectrum of ZnO on a sensor chip.

The Raman spectrum of ZnO displayed the characteristic phonon modes associated with the wurtzite structure, including E_2_(high) mode (440 cm^−1^), A_1_(TO) and E_1_(TO) modes (375–410 cm^−1^) and the A_1_(LO) mode near 570 cm^−1^ (Figure 5). Additional bands observed at 340 cm^−1^ (E_2_ (high) − E_2_ (low) modes) are assigned to the second-order Raman phonon and 1140 cm^−1^ (2LO) may be attributed to second-order Raman processes. These features confirm that the screen-printing and subsequent thermal processing preserved the crystalline wurtzite ZnO phase [56]. Zeolite-overlaid sensors exhibited strong fluorescence under 532 nm excitation, which obscured the underlying ZnO Raman signals. This behaviour is well-known for aluminosilicates and is attributed to electronic transitions associated with framework aluminium and extra-framework cations [57]. While fluorescence prevented detection of ZnO vibrational modes underneath the overlayers, it confirms the presence of the zeolite layer, consistent with the other characterisation findings.

#### 3.1.3. Energy Dispersive X-Ray Spectroscopy (EDX)

EDX was used to verify the elemental composition of the sensors and to confirm the presence of the zeolite overlayers (Table 3). As expected, the ZnO control sensor displayed Zn and O as the dominant elements, confirming ZnO as the principal phase. The measured Zn:O atomic ratio (~60:40) deviates from the ideal 1:1 stoichiometry for ZnO, which is likely due to the limitation of EDX quantification for light elements such as oxygen. In the zeolite-modified sensors, the ZnO signal was significantly attenuated or nearly absent, reflecting the limited penetration depth (typically in the order of 1–10 µm) of the 20 keV electron beam through the zeolite overlayers. This attenuation is consistent with the full overlayer coverage and may indicate that the zeolite layers act as surface-dominant elements in gas interactions.

The presence or absence of Al and Si in the spectra aligned with the expected framework compositions of the zeolites. For high-silica zeolites such as H-ZSM-5 and H-Y, Al was occasionally below the detection limit because Zn, O, and Si dominate the sampled volume, or the section scanned could have contained a small amount, or aluminium was not present in that section. These results are in agreement with the high Si/Al ratios reported for these materials, and they confirm that the zeolites retain their framework composition following the sensor fabrication process. For Na-A, Si, Al, and Na signals were readily detected, supporting successful deposition of chemically distinct zeolite overlayers.

#### 3.1.4. Scanning Electron Microscopy (SEM)

**Figure 6:**
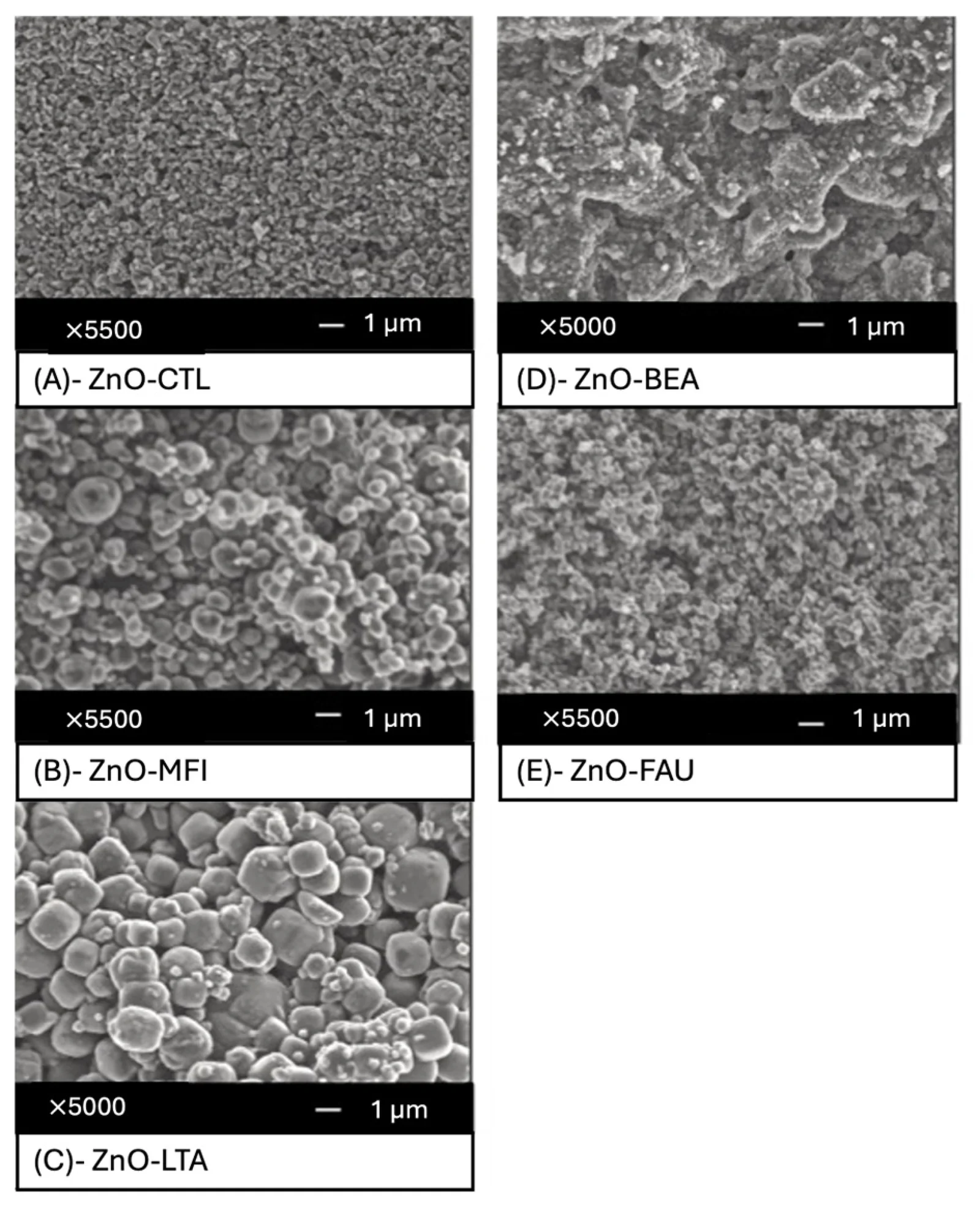
SEM images of (**A**) unmodified ZnO and ZnO with overlayers of zeolite (**B**) MFI: H-ZSM-5, (**C**) LTA: Na-A, (**D**) BEA: H-Beta and (**E**) FAU: H-Y.

Sensor material morphology was studied with Scanning Electron Microscopy (SEM) (Figure 6). SEM images were taken at ×5000, ×5500 or ×6000 magnifications. While the magnifications used limit precise grain size determination, the images confirmed complete and continuous coverage of the ZnO material by the zeolite overlayers.

SEM imaging revealed a uniform granular ZnO morphology in the control sensor (Figure 6A). In the zeolite-modified sensors, the microstructures reflected the characteristic morphologies of each zeolite: H-ZSM-5 grains were both rounded and elongated (Figure 6B); Na-A exhibited well defined cubic particles (Figure 6C); H-Beta (Figure 6D) and H-Y (Figure 6E) displayed much smaller grains that appeared more like agglomerates.

These morphological differences among the zeolites also help explain the varied sensor behaviours seen in later sections below; smaller, more irregular grains (H-Beta, H-Y), may create more tortuous diffusion pathways, whereas the cubic grains may create easier access to the ZnO sensing layer.

### 3.2. Sensor Exposure to an Oxidising Gas

#### 3.2.1. Optimal Operating Temperature

**Figure 7:**
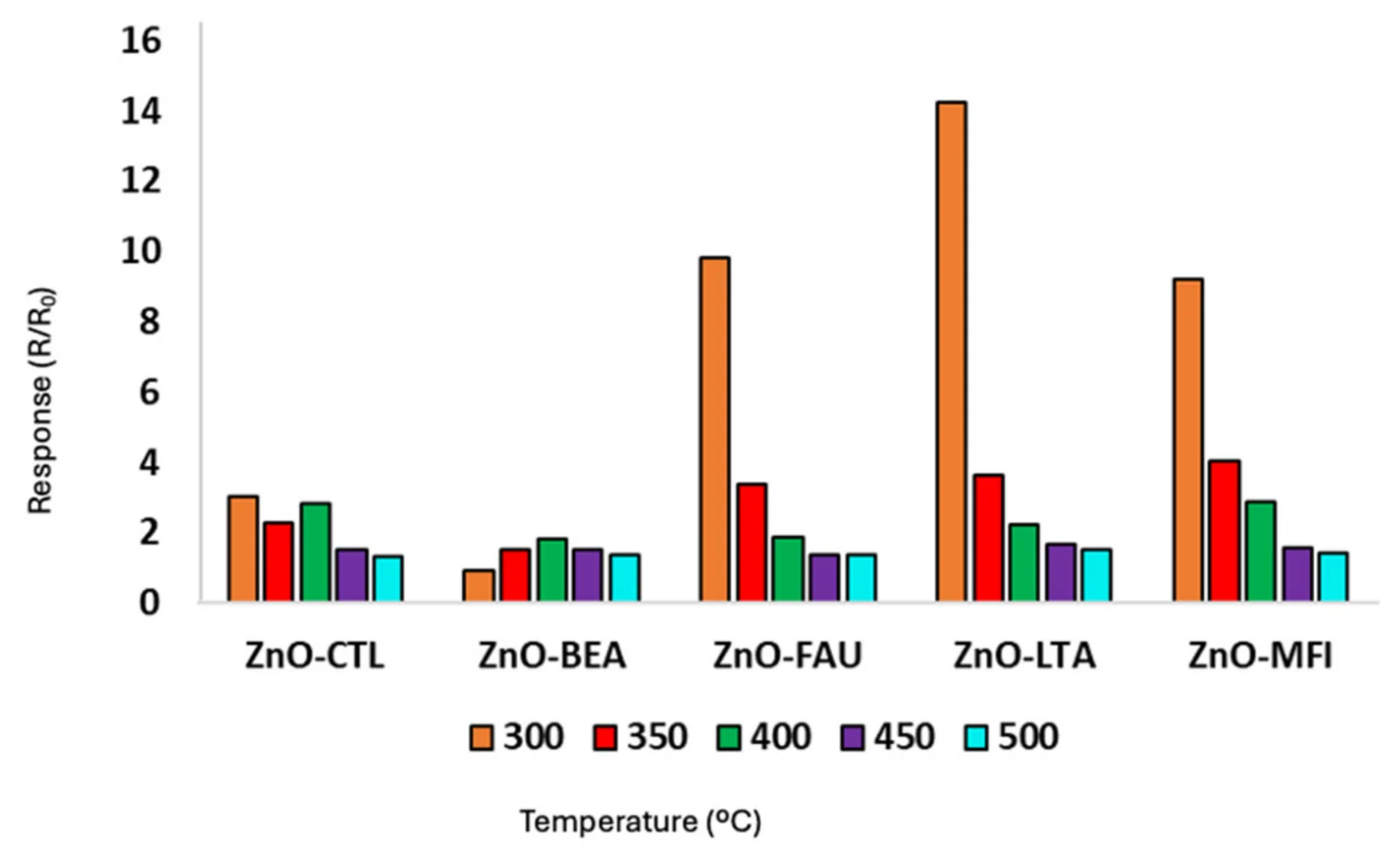
ZnO-based sensor response (R/R_0_) during exposure to NO_2_ (600 ppb) as a function of temperature.

Sensors were exposed to 600 ppb nitrogen dioxide (NO_2_) between 300 and 500 °C to determine the sensors’ optimal operating temperature (Figure 7). As illustrated in Figure 7, sensor responses strongly depended on temperature.

The control sensor as well as sensors modified with zeolites H-Y (ZnO-FAU), Na-A (ZnO-LTA) and H-ZSM-5 (ZnO-MFI) were shown to be most responsive to NO_2_ concentrations at 300 °C, whereas the ZnO-BEA sensor was most responsive to NO_2_ at 400 °C. As seen in Figure 7, increasing temperature generally decreased sensor responses to the gas.

Betty et al. [58] conducted charge carrier transport examinations using DC conductance and impedance studies in an n-type thin film sensing material; they found that the sensitivity to test gases decreased as the temperature incremented, which was the result of fewer oxygen and gaseous species adsorbing at the surface, failing to produce a sizeable resistance change during gas/oxygen interaction.

The above study is in line with what is reported by Korotcenkov [59]; where it is stated that at high temperatures the rates of gas desorption increase and there are fewer adsorbed species available for gas interaction, which leads to lower sensor responses. As reported by Ahlers et al. [60], high temperatures lead to higher reactivity between gaseous species and oxygen and to a limited penetration of gas molecules into the material bulk, which consequently leads to increased rates of gas desorption. In a different report by Korotcenkov [61], it was detailed that at high temperatures the rate of response kinetics is fast, which leads to an equilibrium being reached between the partial pressure of oxygen and the material bulk. At moderate temperatures, the gas will react with the sensitive material. However, due to the diffusion of oxygen into the material bulk being less profound, the chemical composition of the sensing material fails to reach an equilibrium state during the time of gas exposure. At moderate temperatures, redox reactions are thought to take place. At low operating temperatures, chemisorption based on adsorption and desorption processes is thought to prevail [61].

#### 3.2.2. Sensor Responses to Nitrogen Dioxide

**Figure 8:**
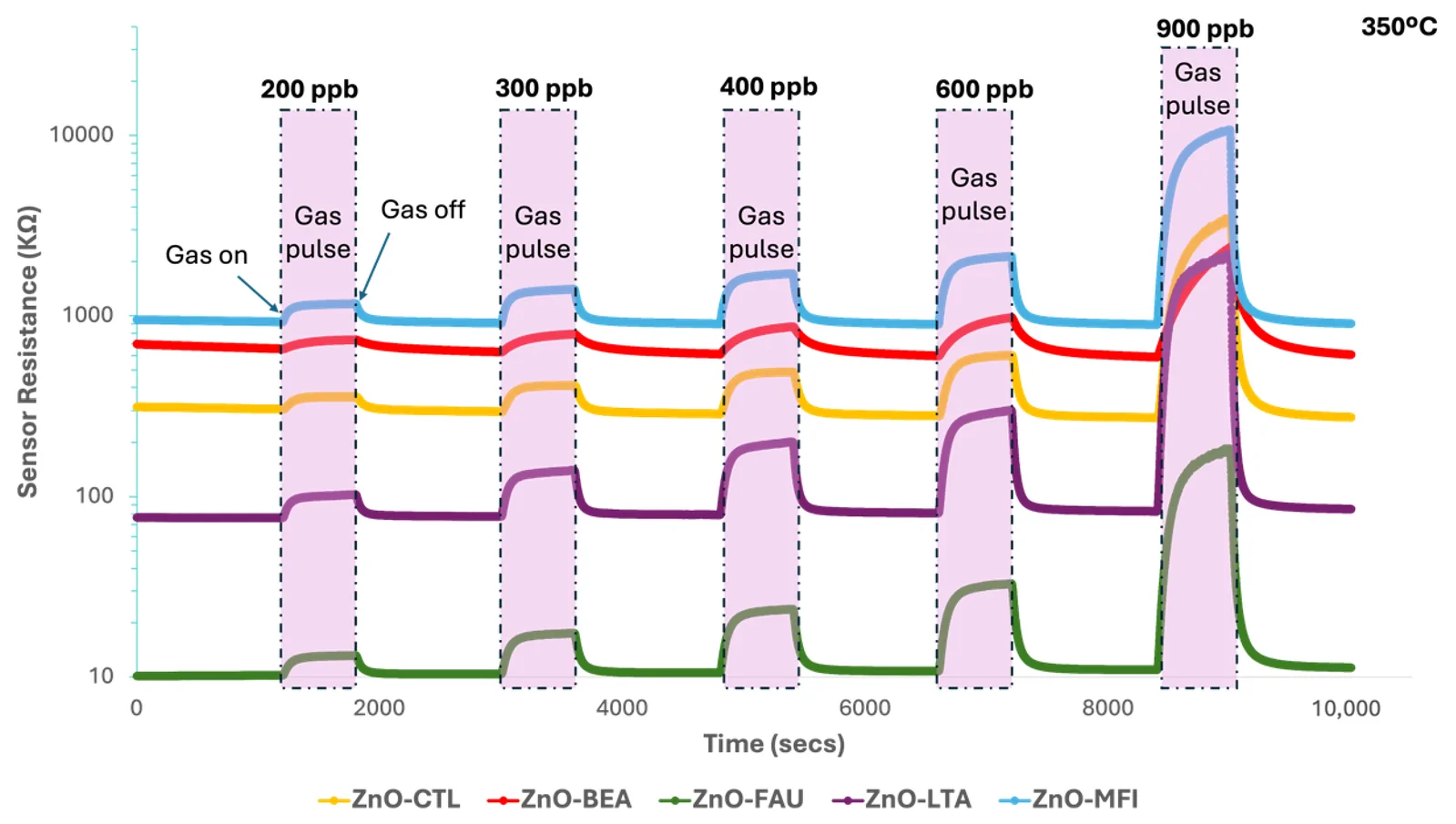
Resistance changes in ZnO-based sensors upon NO_2_ exposure at concentrations of 200, 300, 400, 600, and 900 ppb at 350 °C.

**Figure 9:**
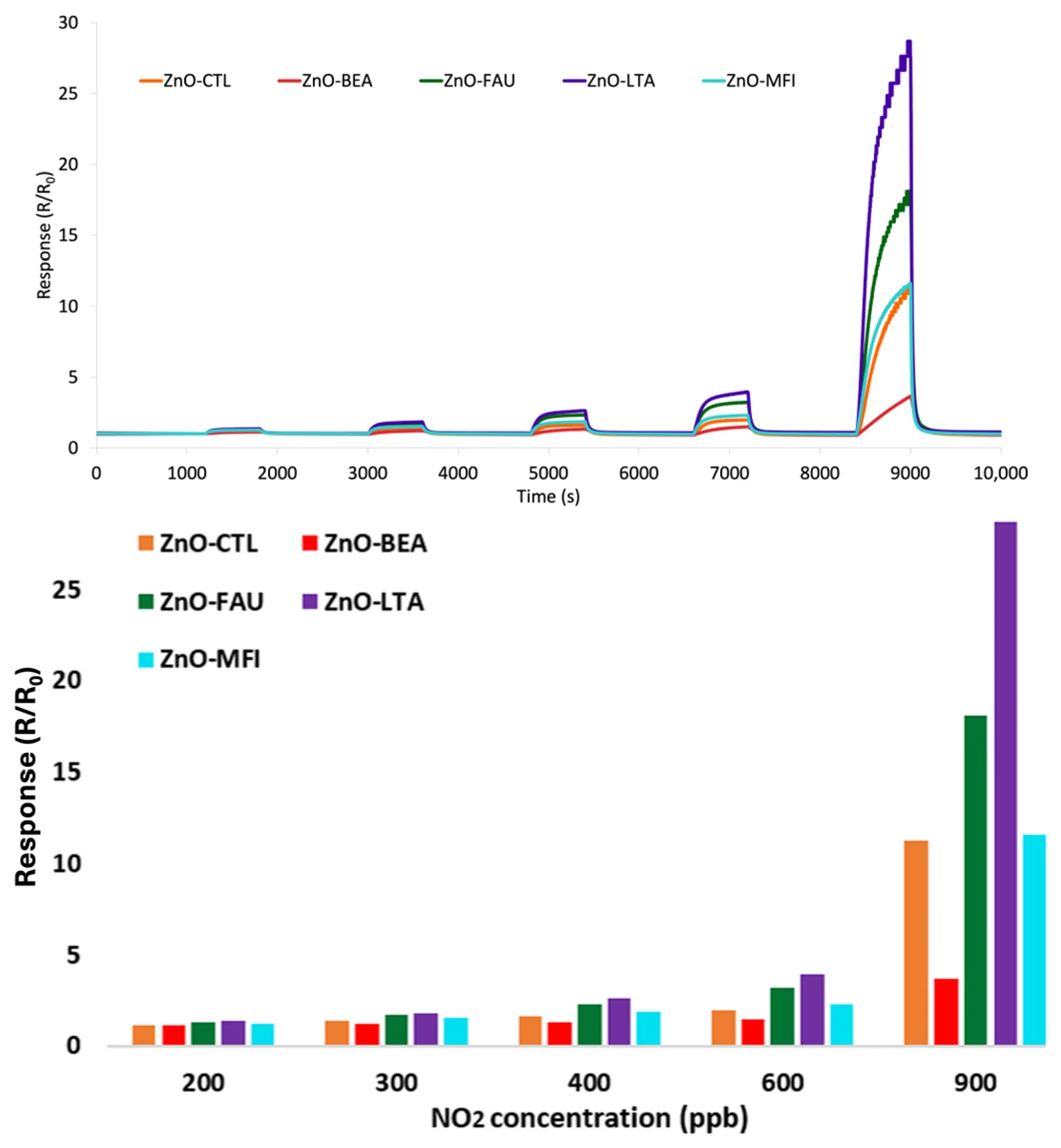
Sensor transients (**top**) and responses (**bottom**) of zeolite-modified ZnO sensors towards NO_2_ concentrations of 200, 300, 400, 600 and 900 ppb at 350 °C.

Sensors were exposed to NO_2_ concentrations ranging between 200 and 900 ppb at 350 °C. NO_2_ concentrations are shown in ppb due to sub-ppm relevance. Sensors consistently increased in resistance when exposed to increasing gas concentrations—as expected for n-type semiconductor materials (Figure 8). Sensors were responsive to NO_2_ at concentrations as low as 200 ppb and improved drastically at 900 ppb (Figure 9).

Surface reactions on n-type semiconductors depend on various factors, such as the surface conductivity of the oxide [62,63], temperature, gas type and concentration [62]. It has been suggested in prior literature that NO_2_ is likely to chemisorb on the sensing material’s surface, leading to subsequent electron withdrawal from the bulk [64].

This then induces further resistance increase, as described by [64,65] and shown in Equation (2) [64].

NO_2_ + e^−^ → NO_2_^−^ (2)

Zeolite incorporation considerably improved sensor responses towards NO_2_. An exception was ZnO-BEA, which showed lower responses than the control sensor. At 350 °C, ZnO-CTL gave a maximum sensor response (R_max_) = ~10 towards 900 ppb NO_2_. Na-A incorporation resulted in a great enhancement (2.8-fold) in sensor response, with R_max_ = 28. ZnO-FAU’s R_max_ = ~18 and ZnO-MFI’s = ~12, whereas ZnO-BEA’s R_max_ = ~4. Peak shapes typically displayed a “shark-finned” appearance, suggesting that transport through the zeolite overlayer influenced the rate at which NO_2_ reached the ZnO sensing layer [64]. In addition to framework-dependent diffusion effects, adsorption of NO_2_ within the zeolite pores may have altered analyte availability at the ZnO interface. The catalytic nature of the zeolites may have also contributed to the formation of surface intermediates or reaction products [64], although this was not directly investigated in the present study.

Sensor response times varied across ZnO-based sensors when supplied with 600 ppb NO_2_ and as a function of temperature. At higher temperatures, sensors responded more quickly (~46–91 s). Particularly at higher temperatures, sensors ZnO-FAU, ZnO-MFI, ZnO-LTA responded more quickly than ZnO-CTL. As detailed previously, at high temperatures the rate of response kinetics is fast, and it is likely that the presence of catalytic zeolite materials further contributes to this behaviour.

**Figure 10:**
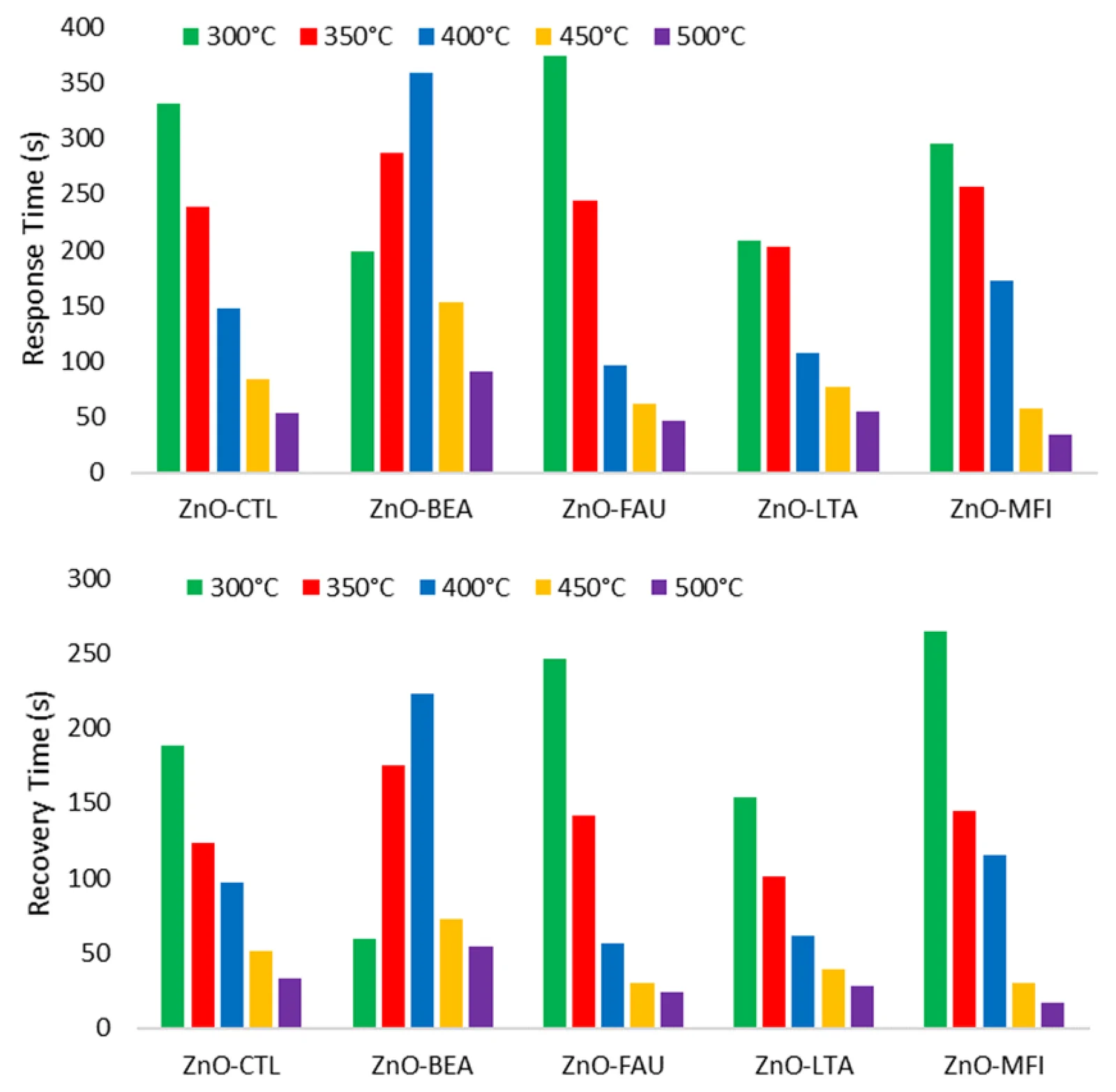
The response time (**top**) and recovery time (**bottom**) of zeolite-modified sensors towards 600 ppb of NO_2_ over a temperature range of 300 °C to 500 °C.

The increase in the operating temperature from 300 to 500 °C reduced the response and the recovery times of the zeolite-modified sensors (Figure 10). The response time for ZnO-MFI was reduced from 296 s to 34 s, whereas the response time for ZnO-LTA was decreased from 208 s to 54 s. This decrease in response time with an increase in operating temperature was observed for every sensor except for ZnO-BEA. This sensor’s response towards NO_2_ was poor, and the sensor did not fully return to its baseline response when exposed to an air ambient. The recovery time for the zeolite-modified sensors was higher than for ZnO which could be due to the additional zeolite layers the gas has to travel through.

### 3.3. Sensor Exposure to Reducing Gases

In the ionosorption model of conduction, during the gas interaction process with a MOS surface, it is thought that reducing gases react and remove oxygen ions from the n-type semiconductor surface. Electrons are then reintroduced into the metal oxide conduction band, resulting in increased charge carrier density and, consequently, a relaxation of the depletion layer. This leads to increased material conductivity [66]. In more recent literature, a surface conductivity model is proposed, whereby changes in sensor conductivity emerge from lattice-O. In the latter model, target gases remove lattice-O from the surface of the sensing material. This then leads to an increase in the surface vacancy concentration and, in turn, an increase in the surface’s conductivity is observed [63].

#### 3.3.1. Optimal Operating Temperature

Sensors were exposed to ethanol, carbon monoxide, propane, and acetone gases. Each sensor was heated to temperatures ranging between 300 and 500 °C to establish the optimal operating temperature.

#### 3.3.2. Sensor Responses to Ethanol

**Figure 11:**
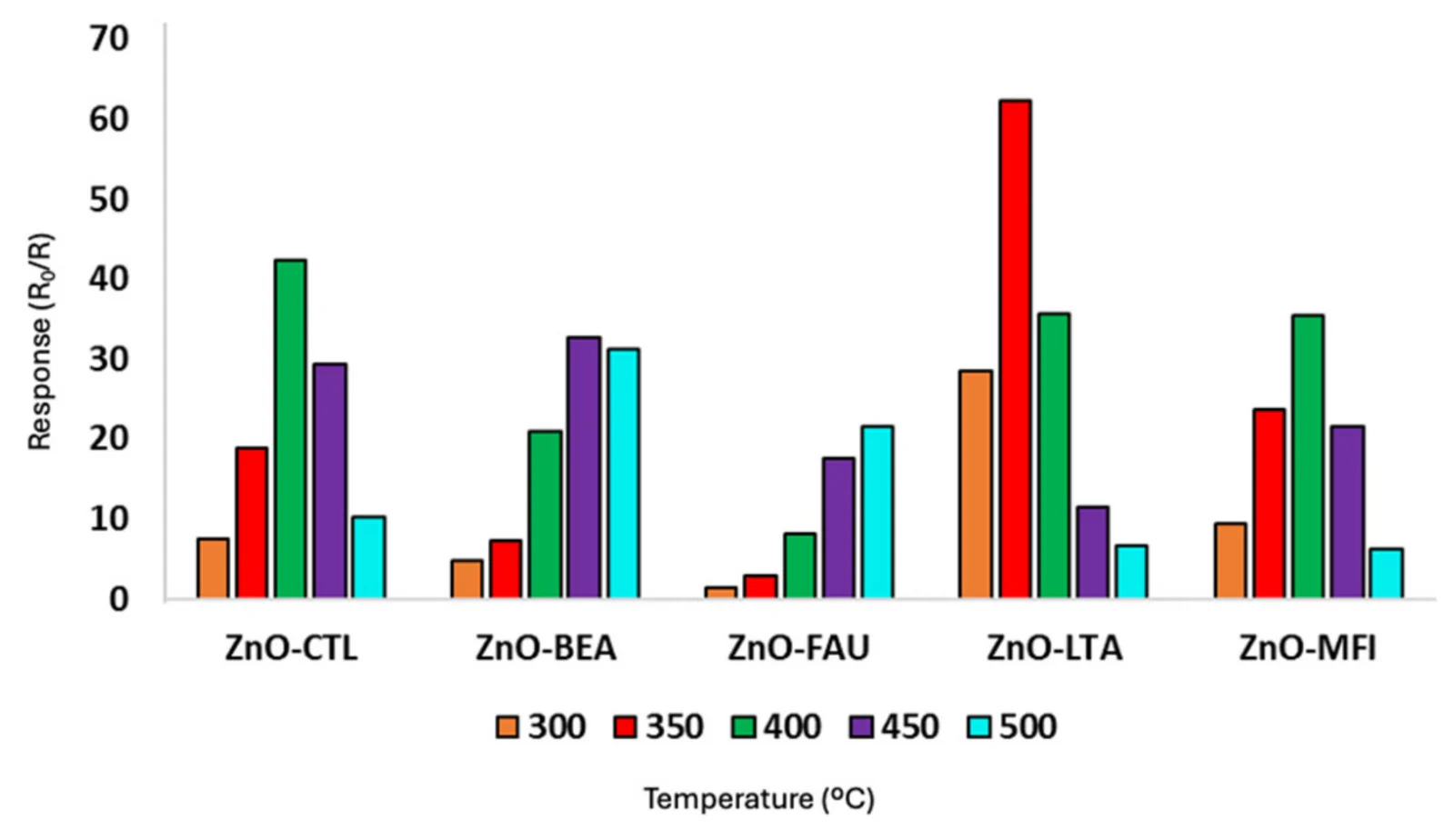
Sensor responses (R_0_/R) to 60 ppm ethanol at temperatures ranging between 300 and 500 °C.

Sensors decreased in resistance upon ethanol exposure, as expected for n-type semiconductors (Figure 11). The optimal operating temperature of zeolite-modified sensors differed from that of ZnO-CTL, which had the highest response at 400 °C (R_0_/R = 42.5); aligned with ZnO’s known high operating temperature [16].

Zeolite overlayers induced notable changes in sensor response when compared to ZnO-CTL (Figure 11). ZnO-BEA displayed low responses at lower temperatures (300 °C and 350 °C) but gave the highest response at 450 °C (R_0_/R = 32.8). Overall, ZnO-FAU displayed lower ethanol responses. This sensor’s response progressively increased with increasing temperature, making 500 °C its optimal operating temperature for ethanol sensing. ZnO-LTA responded most sensitively towards ethanol, with R_0_/R = 62.3 at 350 °C. At higher temperatures, ZnO-LTA sensor responses decreased drastically. ZnO-MFI gave the highest response at 400 °C with R_0_/R = 35.4. This sensor behaved similarly to ZnO-CTL, where the sensor response initially increased with temperature, with 400 °C being the optimal temperature, and then progressively decreased.

Higher ethanol concentrations resulted in increased response magnitudes, with more analyte gas available to undergo a reaction (Figure S1). Response shapes indicate sensors were unsaturated during gas exposure, suggesting longer pulses would have increased response magnitudes. The highest response at 400 °C was obtained by ZnO-CTL (R_0_/R = 42.5). In contrast, sensor ZnO-FAU, seemed less reactive at lower temperatures in relation to other sensors, but still displayed a high response (R_0_/R of 8.1). At 450 °C, ZnO-CTL gave a response of 27.2, whereas ZnO-BEA, ZnO-LTA, and ZnO-MFI responses were R_0_/R = 32, 12 and 21.9, respectively.

Given that electrons are reintroduced into the conduction band of the semiconducting material, one possible reaction that ethanol undergoes with chemisorbed oxygen species on the sensor surface is the dehydrogenation to acetaldehyde. Dehydrogenation of ethanol into CH_3_CHO is given in Equation (3) [67]:

C_2_H_5_OH + O^−^ → CH_3_CHO + H_2_O + e^−^ (3)

CH_3_CHO can undergo further reactions with present oxygen ions to form CO_2_ and H_2_O (Equation (4)).

CH_3_CHO + 5O^−^ → 2CO_2_ + 2H_2_O + 5e^−^ (4)

Another possibility is the dehydration of ethanol to ethylene, which can also undergo further reaction to form CO_2_ and H_2_O (Equation (5)).

C_2_H_5_OH + 6O^−^ → 2CO_2_ + 3H_2_O + 6e^−^ (5)

These two processes can occur on the sensor surface and are dependent on the oxide surface’s acidity. In basic environments, dehydrogenation is favoured, whereas in acidic environments, dehydration is more likely. An increase in operating temperature reduced sensor response times considerably (Figure S2), e.g., ZnO-MFI has a response time of 289 s at 300 °C, which is reduced to 14 s at 500 °C. At higher temperatures the sensor responses will be attenuated due to faster reaction kinetics, which affect gas diffusion by shortening the penetration depth of the gas into the sensing layer [68]. Sensor response times increased with gas concentration, also reported in [69], who investigated ZnO ethanol sensing responses between 350 and 500° and reported longer response times than seen here (510 s for pure ZnO at 400 °C). Sensor recovery times decreased with increasing temperature (Figure S3). Over 300–500 °C, it can be observed that, occasionally, zeolite layers influenced response times in relation to the control sensor, which could be due to a longer time to access the sensitive layer imposed by the zeolite layers.

#### 3.3.3. Sensor Responses to Propane

**Figure 12:**
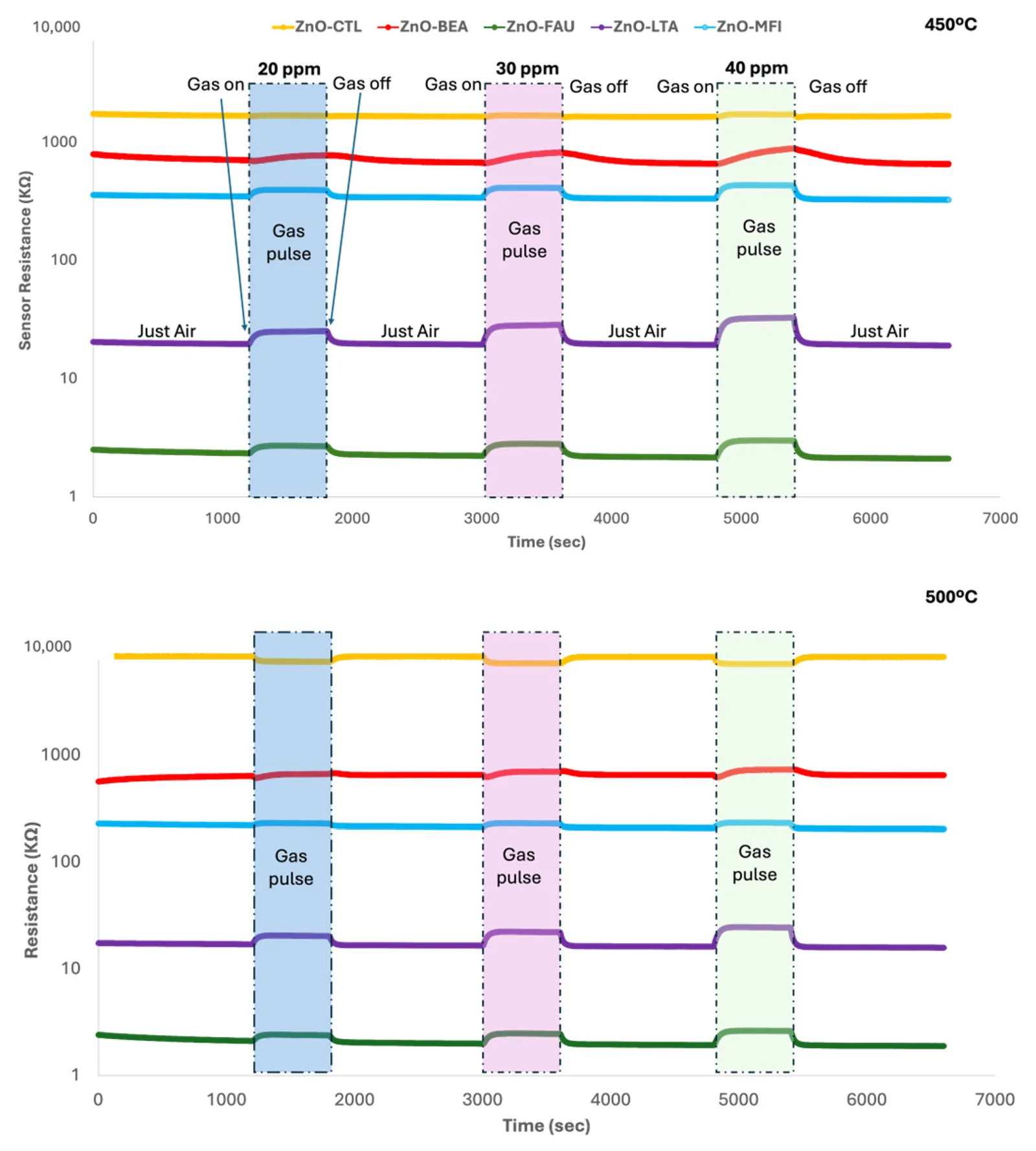
ZnO-based sensor resistance when exposed to 20, 30, and 40 ppm propane at (**top**) 450 °C and (**bottom**) 500 °C.

**Figure 13:**
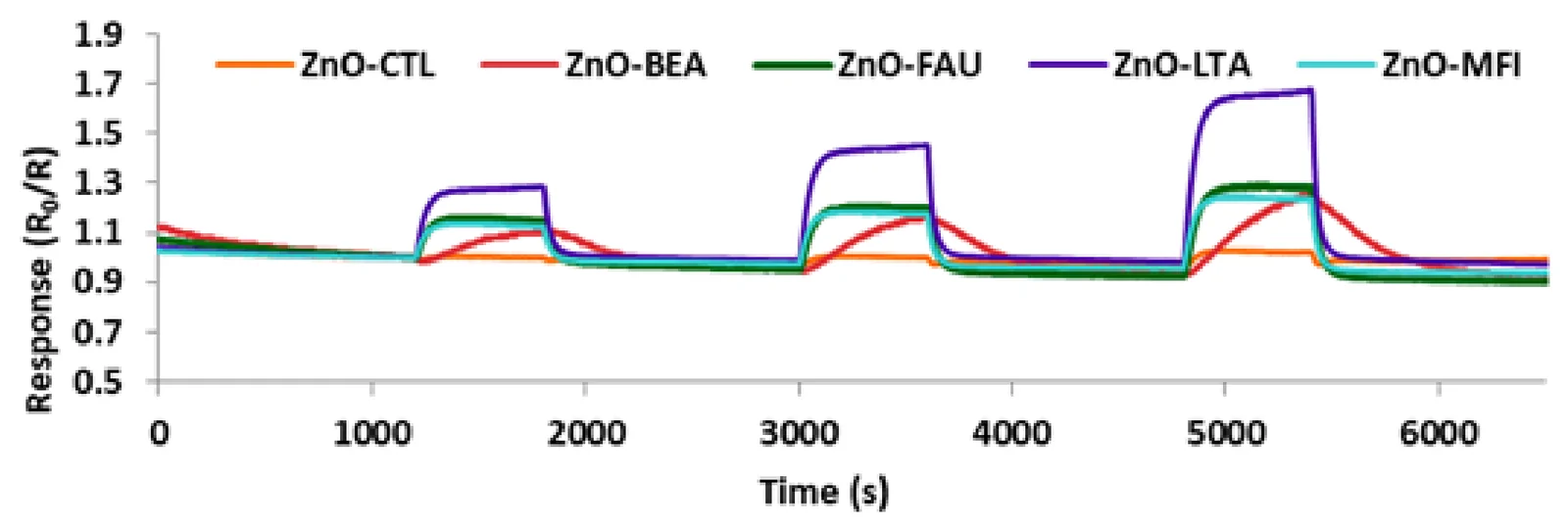

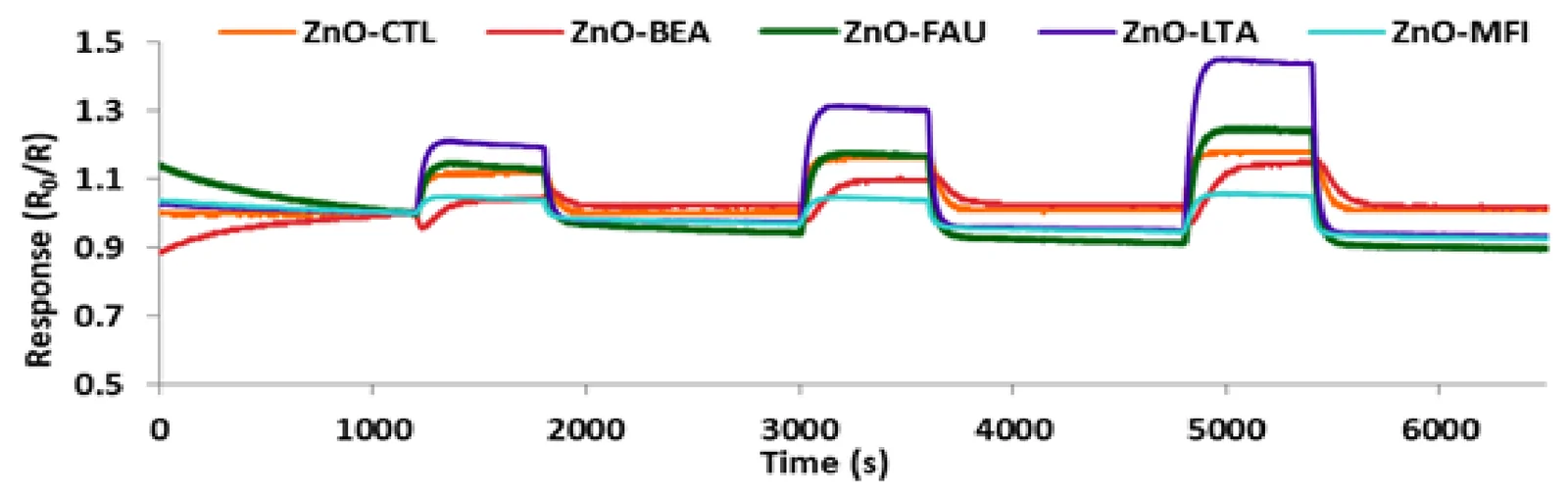
ZnO-based sensor response when exposed to 20, 30, and 40 ppm propane at (**top**) 450 °C and (**bottom**) 500 °C.

Sensors were exposed to 20 ppm, 30 ppm and 40 ppm of propane gas (Figure 12, Figure 13 and Figure S4 for other temperatures).

Most sensors behaved as p-type semiconductors with propane exposure. This behaviour was observed in all zeolite-modified sensors. In the literature, transitions between n-type and p-type-like sensing responses in MOS sensors have been attributed to several factors, including variations in oxygen adsorption, changes in surface band bending, formation of depletion or inversion layers, and the influence of adsorbed species such as humidity or reaction intermediates [70]. However, there is no clear consensus on a single dominant mechanism, particularly in composite systems involving zeolites.

ZnO-CTL showed the expected resistance decrease when exposed to propane at 500 °C. ZnO-CTL exposure to 40 ppm propane resulted in R_0_/R = ~1.7 at 400 °C, indicating ZnO is not ideal for propane sensing.

The reaction mechanism for propane gas on a sensor surface has been reported in [71]. Sensors’ response curves presented a flat top, indicating saturation; thus, longer gas pulses would not improve sensor responses.

#### 3.3.4. Sensor Responses to Carbon Monoxide

**Figure 14:**
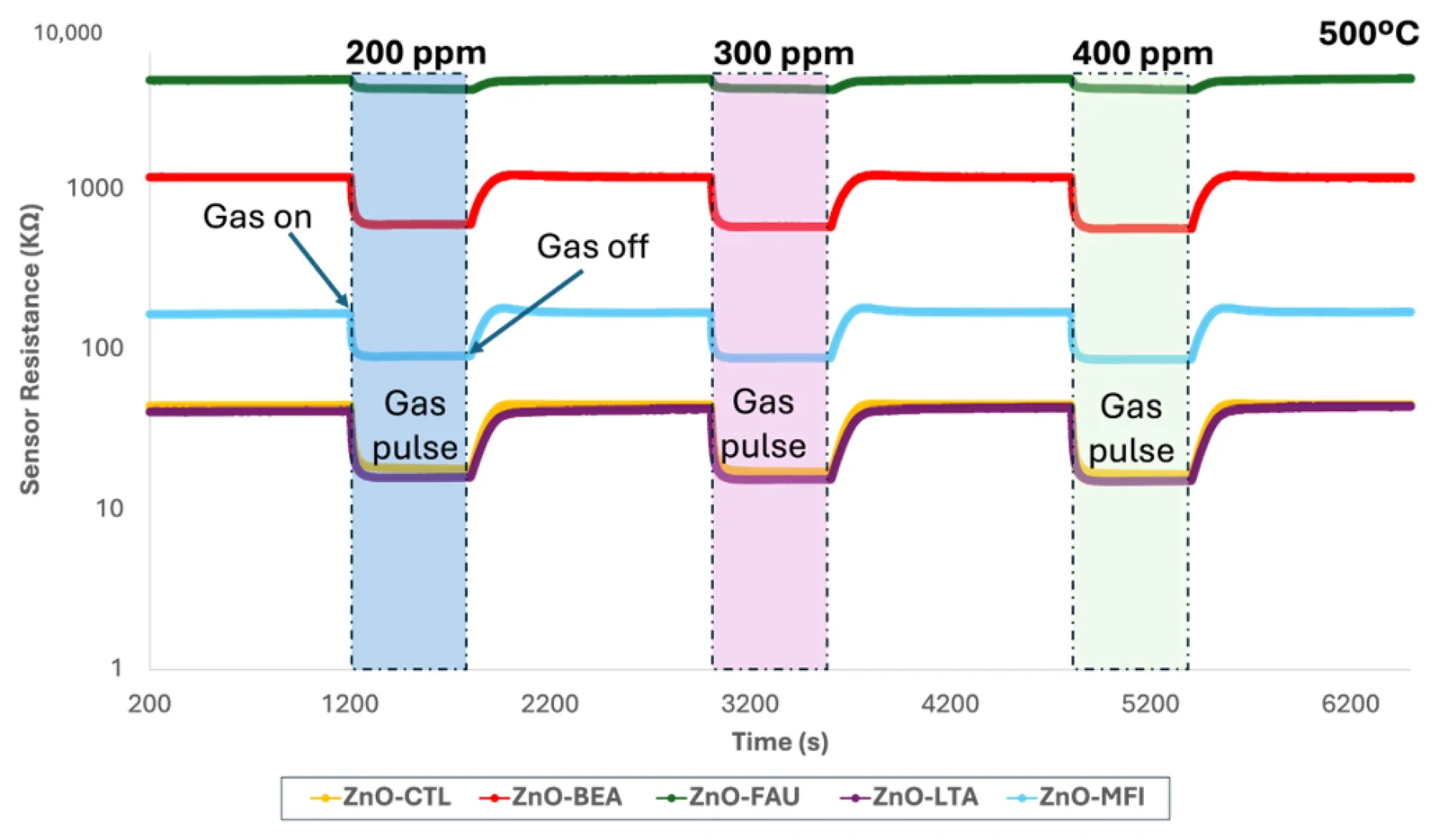

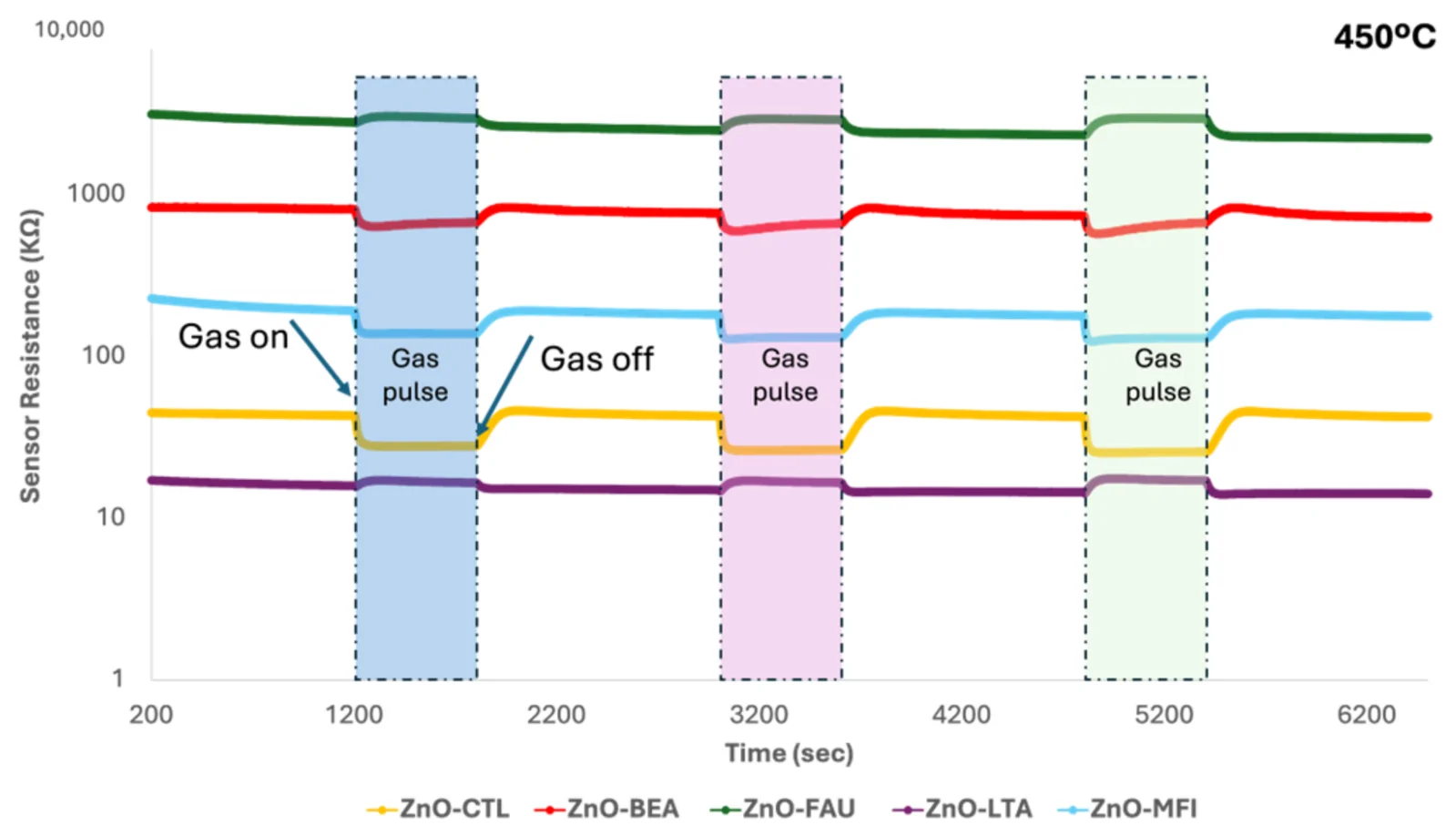
ZnO and zeolite-overlayed sensor resistance when exposed to 200, 300, and 400 ppm CO between 450 and 500 °C.

**Figure 15:**
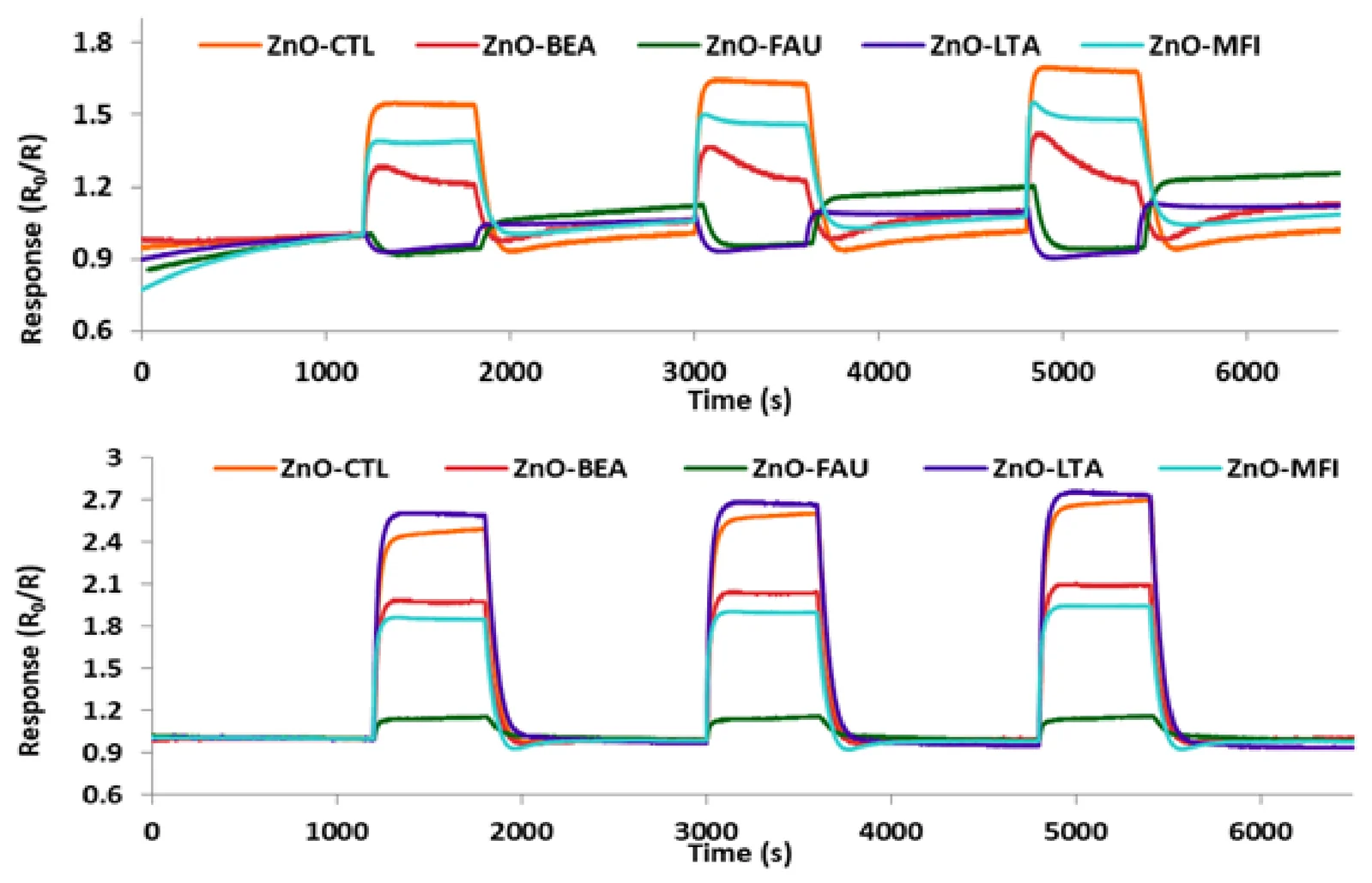
Zeolite-modified ZnO sensor response towards 200, 300 and 400 ppm CO at operating temperatures of 450 °C (**top**) and 500 °C (**bottom**).

Sensors were exposed to 200, 300 and 400 ppm CO at 450 °C and 500 °C (Figure 14 and Figure 15). Resistance changes at lower temperatures have been presented in Figure S5 and response patterns at other temperatures have been included in Figure S6. Overall, sensor responses towards CO were low across these temperatures, with most responses close to baseline resistance. Increases in gas concentration induced minor increases in sensing response. Sensors responded with an increase in resistance upon CO exposure. An exception was seen in ZnO-BEA at 300 °C and 350 °C (Figure S5). Only at 500 °C did all sensors provide expected behaviour of n-type semiconductors with reducing gas exposure. At 450 °C, for instance, ZnO-FAU and ZnO-LTA sensors exhibited an anomalous resistance increase upon exposure to CO, which resembled a p-type response. In contrast, ZnO-CTL, ZnO-MFI, and ZnO-BEA sensors displayed the expected n-type response upon exposure to the gas (resistance decrease). This behaviour may be explained by temperature-dependent surface inversion, a mechanism explained in [72] for metal oxide semiconductors exposed to strong oxygen adsorption—see also the explanation provided above for a similar n-type inversion to propane.

The contrasting sensing behaviour observed for ZnO-FAU (H-Y) and LTA (Na-A)-based sensors compared to other sensors suggests that the zeolite component plays a significant role in modifying the sensing mechanism. Differences in pore architecture, cation composition, and adsorption properties are expected to influence gas diffusion, oxygen species, and the local chemical environment at the ZnO–zeolite interface.

In the present case, the enhanced adsorption capacity or confinement effects of FAU and LTA frameworks, respectively, may promote the retention of reactive species. This may lead to the observed reversal in sensing response at intermediate temperatures. At higher temperatures, increased desorption and faster surface kinetics likely restore the conventional n-type behaviour. However, further investigation would be required to unambiguously determine the underlying mechanism responsible for this behaviour. Poor sensitivity could be related to CO_2_ gas production—e.g., Kumar et al. [73] exposed a Ni-ZnO sensor to CO_2_ concentrations in the 500–2000 ppm range, attaining responses of 1.5 to 500 ppm of gas. Van Hieu et al. [74] exposed ZnO nanowires to 200 ppm of CO, obtaining a response (R_0_/R) of ~2.

Sensor response and recovery times to 400 ppm CO were investigated at 500 °C (Figure S7) but have not been reported at other temperatures due to a general baseline drift, which is common in MOS devices, but can self-correct over time [75].

#### 3.3.5. Sensor Responses to Acetone

Acetone concentrations tested were 10 ppm, 20 ppm, 30 ppm, 40 ppm and 60 ppm and at operating temperatures of 300–400 °C. ZnO-LTA-based results have not been included due to a problem with the sensor port.

**Figure 16:**
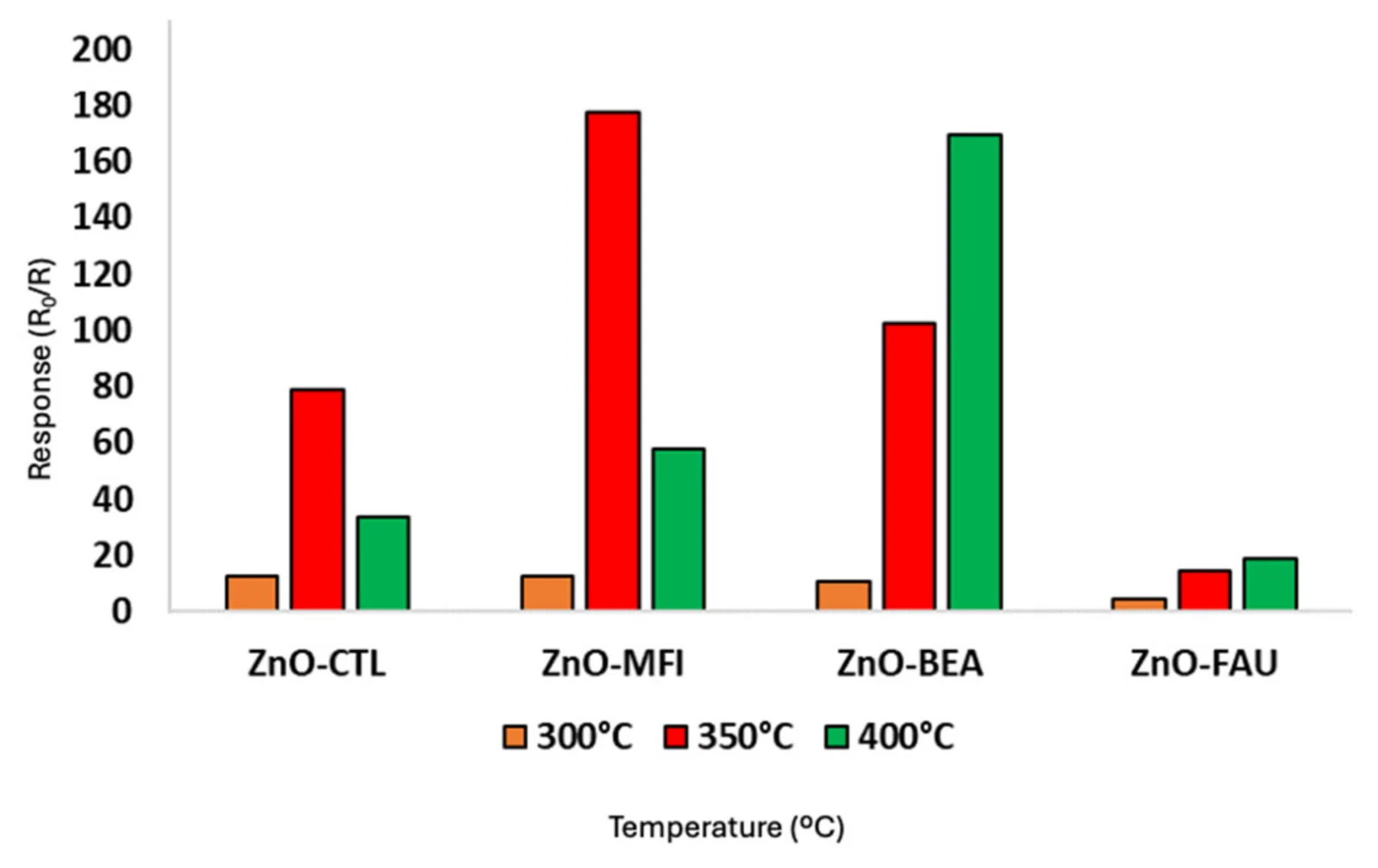
Sensor responses of zeolite-modified sensors towards 60 ppm acetone as a function of increasing temperature.

**Figure 17:**
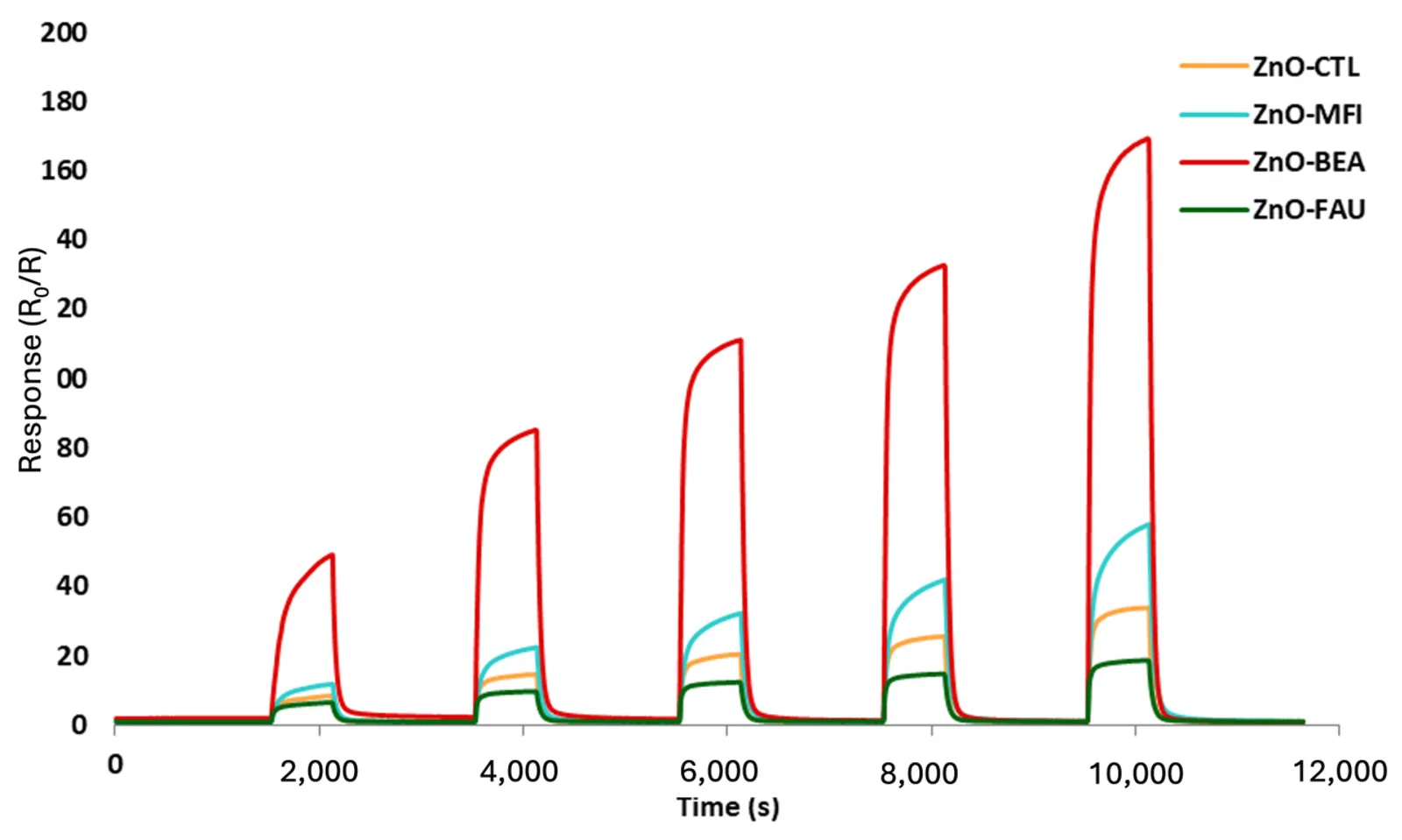
Conductive gas responses for zeolite-modified ZnO sensors to 10, 20, 30, 40 and 60 ppm of acetone at 400 °C.

Figure 16 shows the sensors’ response to 60 ppm acetone across various operating temperatures. The sensors’ resistance decreases upon exposure to acetone, and response magnitudes increase with concentration. The highest responses were obtained by ZnO-MFI with a response of R_0_/R = 177.4 (±26.1) at 350 °C, followed by ZnO-BEA at 400 °C R_0_/R = 169.5 (±4.1) when exposed to 60 ppm. ZnO-FAU displayed considerably lower responses compared to other zeolite-modified sensors at all temperatures. Sensors displayed high affinity towards acetone (Figure 17). The lowest sensor responses were seen in ZnO-FAU, which saturated quickly, and increased from R_0_/R = 6.5 (±0.8) at 10 ppm to R_0_/R = 18.9 (±0.8) at 60 ppm.

Sensor response and recovery times to 60 ppm acetone are displayed in Figure S8 and Figure S9, respectively. Increasing temperature resulted in decreased response times.

ZnO-CTL generally displayed one of the highest response times next to ZnO-MFI; some zeolites might encourage gas diffusion to the sensing material, leading to longer recovery times if the gas displays particular affinity for that zeolite.

It is thought that the reaction that occurs at the sensor atmosphere upon reaction with acetone is as shown in Equation (6) [76]:

CH_3_COCH_3_ + 8O^−^ (8O^2−^) → 3CO_2_ + 3H_2_O + 8e^−^ (16e^−^) (6)

### 3.4. Zeolite Incorporation Effects

#### Exploiting Zeolite Properties for Gas Detection

The silicon to aluminium ratio (Si/Al) influences zeolite behaviour as the aluminium content controls zeolite hydrophobicity, catalytic character and acidity [35,77,78]. These were capabilities that were explored with ZnO as, to the best of our knowledge, they have not yet been reported in the literature.

Zeolites with a low ratio (1–2) and an intermediate ratio (~2.5–10) are hydrophilic, whereas zeolites with high Si/Al ratios (>10) are hydrophobic (Table 4) [79]. The authors anticipated that these properties would attract molecules according to their polarity and this would translate into higher or lower sensor responses towards some vapours. Utilising zeolite materials with higher surface area increases the surface reactive sites on the sensor surface thus providing further opportunities for molecule adsorption [35]. However, it is worth noting that the accessible surface area following screen-printing and thermal processes were not directly measured in this study. Other studies have suggested that differences in zeolite frameworks can help in providing size and shape discrimination in similar gases [80,81].

Nitrogen Dioxide: N-O bond distance is 1.19 Å. Thus, NO_2_ could easily pass through all four zeolite pores. When using ZnO-CTL as the base material, low Si/Al zeolites such as Na-A resulted in enhanced sensor responses to NO_2_. Although smaller responses were obtained when using FAU-type zeolites, intermediate zeolites also demonstrated NO_2_ affinity, thought to be due to the high adsorption capacity for NO_2_ due to their large pore sizes. High-silica sensors gave poorer NO_2_ responses. The literature suggests that dipole–dipole interactions can create an attraction between NO_2_ and zeolite Na-A’s surface, which can help in this gas’s adsorption onto the sensor surface [64]. It is thought that this zeolite’s high surface area (Table 4) creates increased regions for gas interaction. In contrast, the lower NO_2_ responses observed for the sensor containing H-Beta suggest that sensing behaviour was affected not only by the ability to diffuse through the zeolite’s pores.

Ethanol: A kinetic diameter of *ca*. 4.5 Å is thus smaller than the zeolite pores used here. It may therefore diffuse easily and reach the sensing material, adsorbing onto and reacting with zeolite active sites during diffusion. Ethanol’s higher affinity towards the zeolite Na-A surface could be explained due to its hydrophilic nature [32], and the smaller pores of Na-A and H-ZSM-5 may have created more suitable environments for ethanol adsorption. In contrast, the current findings suggest that the zeolites that had larger pores did not lead to the same high affinity towards this gas.

Propane: Has a kinetic diameter of ~5 Å; thus, its diffusion through ZnO-LTA would be hindered. Furthermore, propane adsorption on a hydrophilic zeolite is unfavourable. The size constraints could lead to diffusion via cracks in the zeolite structure instead of the zeolite pores. Ref. [78] suggests that water formation as part of reaction processes may lead to its adsorption on a hydrophilic zeolite or inside the walls of inter-crystalline voids. This could explain why sensors responded to propane but does not explain the p-type response observed.

Acetone: Sensor responsiveness in H-Y, H-ZSM-5, and H-Beta towards acetone could be attributed to several factors. It is possible that due to steric effects, acetone could not penetrate effectively into H-ZSM-5’s pores; thus, resulting in smaller response magnitudes when compared to ZnO-BEA, and also in comparison to ethanol sensing [36]. Although this is not confirmed in this study, the larger pore sizes in H-Y may have resulted in less effective or weaker interactions with acetone.

Carbon Monoxide: The flattened sensor response suggests that the sensors effectively catalysed the reaction to carbon dioxide, to which the sensor materials do not appear to be very responsive [36]. Furthermore, the observed shift in n-to-p-type behaviour may have been due to the formation of water as a reaction product, and a reasonable interpretation is that the incorporation of zeolites may have contributed to this by accelerating or fully combusting the vapour into CO_2_, as this behaviour was not observed with the control sensor at 450 °C.

## 4. Implications of Findings and Concluding Remarks

Sensors were exposed to various target gases relevant to medical, environmental, safety, and forensic fields. All sensors, including ZnO-CTL, were responsive to the gases tested. Generally, sensors’ responses were enhanced upon gas concentration increase. It is worth noting that zeolite-modified sensors did not always provide an increased sensor response when compared to the control sensor. Some sensors provided remarkably improved responses when compared to the unmodified ZnO-based sensor. Sensor response and recovery times were often improved in these sensors, albeit not always at the lower temperature end, in line with what other studies have reported [47,61]. Nevertheless, it is promising that integration into a sensor array led to different response patterns to investigated gases, providing a first step towards achieving effective gas discrimination in the future.

Sensors were responsive to 200 ppb NO_2_, which suggests that through further modification of the sensors, even lower NO_2_ concentrations could be achieved. In the future, it would be worth exploring nano-powdered ZnO and metal-doped materials to increase sensitivity towards this gas and testing if responsiveness is possible at even lower operating temperatures. In relation to acetone, sensors were highly responsive to 10 ppm of gas. This means that these sensors could presently be used to monitor acetone leaks. These results offer promise to target even lower gas concentrations, which are required in the medical field, where breath concentrations ~1 ppm are targeted [82].

ZnO–zeolite admixtures have previously significantly improved sensor sensitivity and discrimination capabilities towards ethanol [69]. Two studies reported hydrothermal growth of zeolites on ZnO, but these fabrication methods are more complex and time consuming than screen-printing; namely, zeolite A, was hydrothermally grown on ZnO to produce low-temperature Cu-doped ZnO nanostructures for the detection of N_2_O [83], and hydrothermal growth of ZIF-8 on screen-printed ZnO was used to detect hydrogen, but it lacked sensitivity to acetone and ethanol [84]. More recently, spin-coating was used to create MFI-encapsulated Pd-PdO clusters as ZnO overlayers for NO_2_ sensing [85], but this sensor was non-responsive to other target gases. Our sensors responded more favourably to ethanol and acetone than other ZnO-based zeolite-modified sensors (Table S1). However, it appears that admixtures may be a preferred method to target NO_2_.

There are a number of limitations that ought to be acknowledged in this study. Sensors ought to be exposed to mixed-vapour environments to understand their performance under these conditions and suitability in operational contexts. The vapour concentration ranges tested in this study were selected based on the cylinder concentrations available and to enable comparison of different sensor architectures rather than to establish minimum detection limits for environmental, medical, or forensic applications. Furthermore, the proposed sensing mechanisms are based on the zeolites’ characteristics and reported literature studies, and direct measurements of adsorption intermediates or reaction pathways were not conducted. Further studies that incorporate long-term stability assessments, more advanced surface characterisation, and exploration of various zeolite layer thicknesses would provide additional insight into the mechanisms driving framework-dependent sensing behaviour.

In conclusion, the results of this study provide valuable new insights for the design of zeolite–MOS sensor arrays. Pore size and framework structures can be used to control gas access and induce vapour discrimination [81], with small-pore, hydrophilic zeolites (e.g., Na-A) enhancing responses to small polar gases such as NO_2_, while medium- and large-pore zeolites may enable diffusion of larger molecules. Literature suggests that Si/Al ratio and surface chemistry play an important role in gas–zeolite interactions, where hydrophilic frameworks favour polar analytes and high-silica, acidic zeolites promote interactions with less polar species and enable catalytic transformations [32,77,78]. Overall, this study underscores the importance of continuing to understand and exploit zeolite materials as modification agents of metal oxide semiconductors for gas detection, to create systems that are suitable for real-world applications.

## Figures and Tables

**Table 1:** Sensor names, materials, and number of printed overlayers.

Sensor Name	Sensing Material	Overlayers	Number of Layers
ZnO-CTL	ZnO	None	5
ZnO-MFI	ZnO	H-ZSM-5	5 + 3
ZnO-LTA	ZnO	Na-A	5 + 3
ZnO-BEA	ZnO	H-Beta	5 + 3
ZnO-FAU	ZnO	H-Y	5 + 3

**Table 2:** Sensor type and Scherrer sizes (nm).

Sensor Type	Crystallite Size (nm)
ZnO-CTL	32
ZnO-BEA	32
ZnO-MFI	34
ZnO-FAU	35
ZnO-LTA	47

**Table 3:** Atomic percentage of sensor materials using EDX.

Sensor	Elemental Atomic Percentage				
	Zn	O	Si	Al	Na
ZnO-CTL	60.0	39.9	0	0	0
ZnO-MFI	0.4	45.4	55.0	0	0
ZnO-BEA	0	39.5	60.5	0	0
ZnO-LTA	0	53.1	17.8	16.8	12.3
ZnO-FAU	0	46.2	42.2	11.6	0

**Table 4:** Zeolite properties, pore structures, and BET surface area (m^2^/g). Note that the BET values provide information on the surface area characteristics of the zeolite powders, and not of the finalised sensor structure.

Material	Material Details	Pore Sizes (Å)	Powder BET Surface Area (m^2^/g)	Best-Matched Analytes	Observed Sensing Behaviour
H-Beta	Large pores + high acidity and surface area, hydrophobic	12-membered channel system | 5.6 × 5.6 Å and 6.6 × 6.7 Å	663.1	Acetone	Highest acetone response at 400 °C
H-ZSM-5	Medium pores, acidity, hydrophobic	3D 10-membered ring with straight and zig-zag channels | 5.6 Å	382.6	Acetone, ethanol	Strong vapour sensing
H-Y	Large cages, hydrophobic	12-membered ring 3D 6-ring openings (2.6 Å) | super-cage 7.5 Å	429.1	NO_2_	Moderate NO_2_ enhancement
Na-A	Small, hydrophilic pores	8-membered oxygen ring | 4 Å	573.2	NO_2_, ethanol	Strong NO_2_ enhancement at 300 °C
ZnO	-	-	2.79	General	Unselective

## Data Availability

Data can be made available upon request.

## Supplementary Materials

The following supporting information can be downloaded at https://www.mdpi.com/article/10.3390/s26206364/s1, Figure S1: Conductive response (R_0_/R) to the exposure of 20, 40, 60 ppm of ethanol at an operating temperature of 400 °C for ZnO sensors (top) and sensor maximum response against three gas concentrations at the same operating temperature (bottom); Figure S2: The response time (s) of zeolite modified sensors towards 60 ppm of ethanol over a range of temperatures from 300 °C to 500 °C.; Figure S3: The recovery time (s) of zeolite modified sensors towards 60 ppm of ethanol over a range of temperatures from 300 °C to 500 °C; Figure S4: Response towards 40 ppm of propane as a function of increasing operating temperature; Figure S5: ZnO-based sensor resistance to 200, 300, and 400 ppm Carbon Monoxide between 300 and 400 °C.; Figure S6: ZnO and zeolite-modified sensors response towards 200, 300, and 400 ppm Carbon Monoxide between 300 and 400°C; Figure S7: Response and recovery times of ZnO sensors towards carbon monoxide gas with different zeolite overlayers at 500 °C. t_res_ refers to the sensor response time (s) and t_rec_ to the sensor recovery time (s); Figure S8: The response time of zeolite modified sensors towards 60 ppm of acetone over a range of temperatures from 300 °C to 400 °C; Figure S9: The recovery time of zeolite modified sensors towards 60 ppm of acetone over a range of temperatures from 300 °C to 400 °C; Table S1: Comparison of sensor performance in present paper to other published studies on zeolite-modified ZnO.

## Abbreviations

The following abbreviations are used in this manuscript:

MOS	Metal Oxide Semiconductor
SEM	Scanning Electron Microscopy
XRD	X-ray Diffraction
EDX	Energy Dispersive X-ray Spectroscopy
BET	Brunauer–Emmett–Teller
