# Supplementary material for: Comparative Study of Zeolite Overlayers on ZnO Gas Sensors: Effects of Framework Structure on Sensing Performance
Source: Sensors (Basel). 2026 Oct 9;26(20):6364. doi: 10.3390/s26206364 (PMC13708887; doi:10.3390/s26206364)
Supplement: Supplementary file 1 [file sensors-26-06364-s001.zip › sensors-4460151-supplementary.pdf]

Supporting Information

**Justification behind gas selection in this study:**

**Nitrogen dioxide** is an environmental pollutant that has serious effects on human health and the environment. Current air quality standards suggest that less than 60 ppb $NO_2$ averaged over 1 hour is considered 'good' air quality, whereas between 120 and 180 ppb $NO_2$ averaged over an hour is considered 'poor' air quality. $NO_2$ has also been used as a detection marker for explosive materials. **Acetone** is a chemical solvent extensively used in laboratories and industrial settings. It has been reported that long-term exposure to acetone can affect the central nervous system and lead to respiratory and kidney disease. The recommended exposure limit to acetone is 250 ppm over a 10-hour work shift period [18]. Acetone also serves as a non-invasive biomarker for diabetes; a concentration in breath above 1 ppm is seen in diabetic patients, whereas a concentration below 1 ppm is observed in non-diabetic patients [31]. Acetone has also been reported as a recreational drug. **Ethanol** is a solvent extensively used in a range of industries such as the food and pharmaceutical industries and in the illegal manufacture of drugs. Its detection is important to monitor leaks or its concentration in air due its high flammability. **Carbon monoxide** detection is important to monitor indoor air quality, particularly in domestic settings, as it can cause accidental poisoning. The recommended exposure levels should not exceed 200 ppm for 15 min over an 8-hour shift. **Propane** is a highly flammable gas, the detection of which is important to monitor gas leaks. In addition, propane has also been reported as an inhalant.

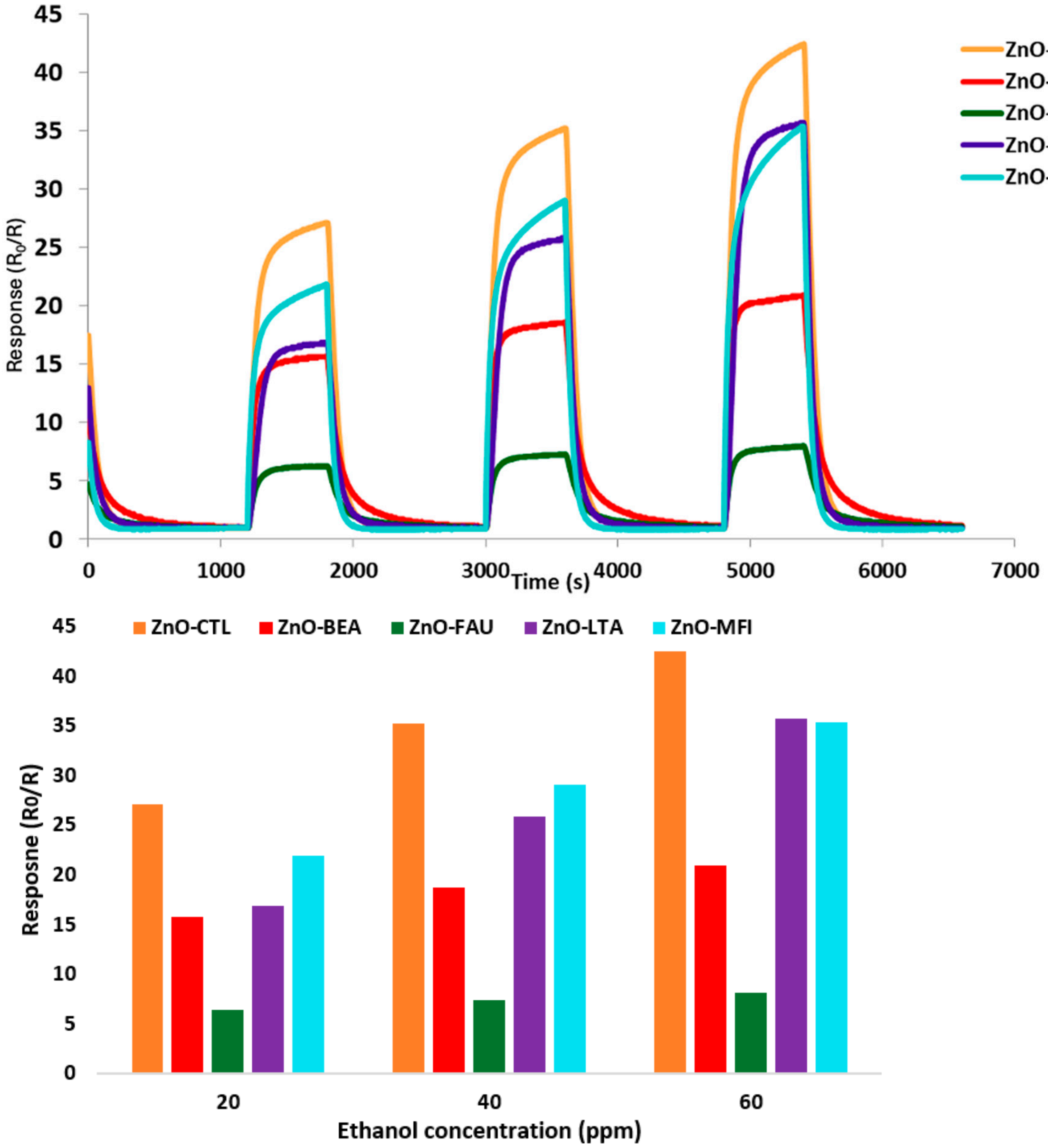


**Figure S1 - Conductive response ($R_0/R$) to the exposure of 20, 40, 60 ppm of ethanol at an operating temperature of 400°C for ZnO sensors (top) and sensor maximum response against three gas concentrations at the same operating temperature (bottom).**

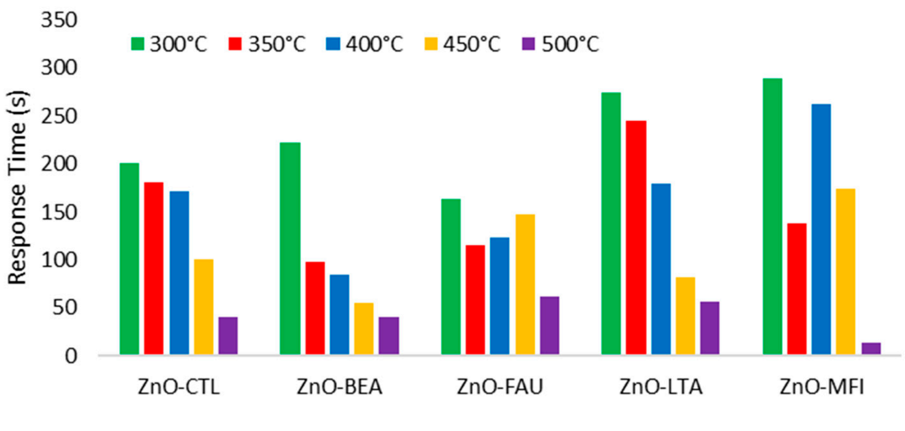

**Figure S2 - The response time (s) of zeolite-modified sensors towards 60 ppm of ethanol over a range of temperatures from 300°C to 500°C.**

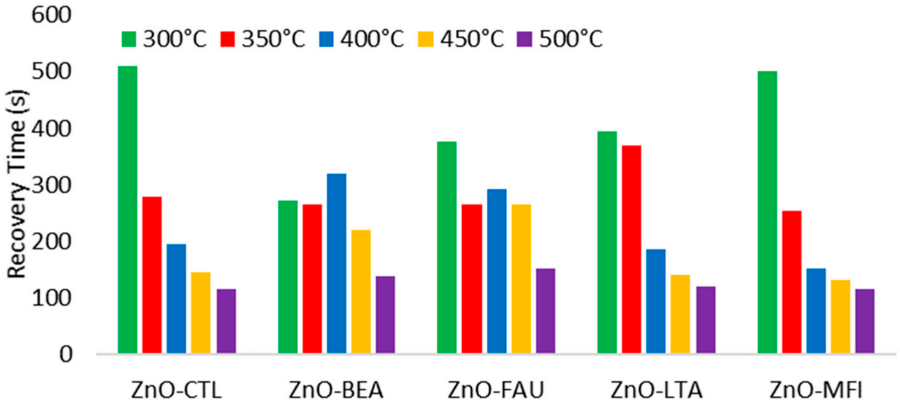


**Figure S3 - The recovery time (s) of zeolite-modified sensors towards 60 ppm of ethanol over a range of temperatures from 300°C to 500°C.**

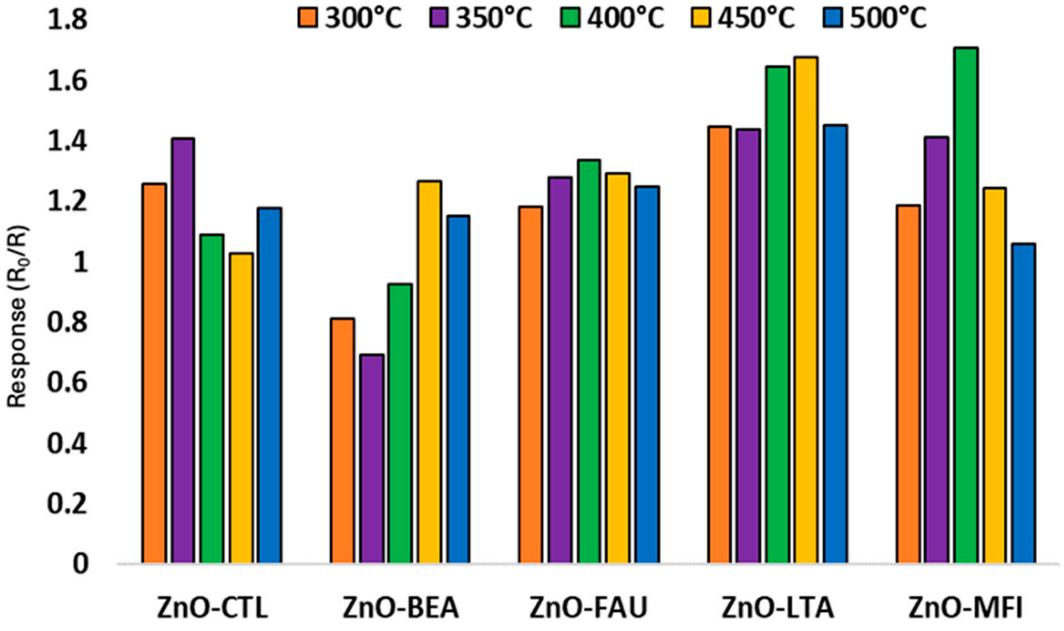


**Figure S4 - Response towards 40 ppm of propane as a function of increasing operating temperature.**

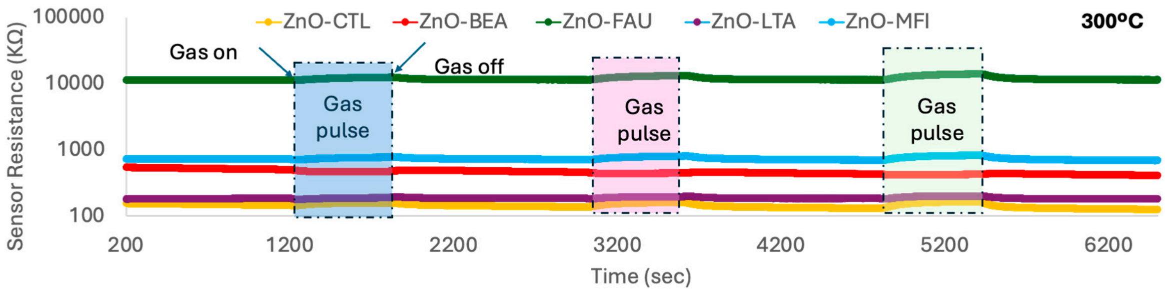

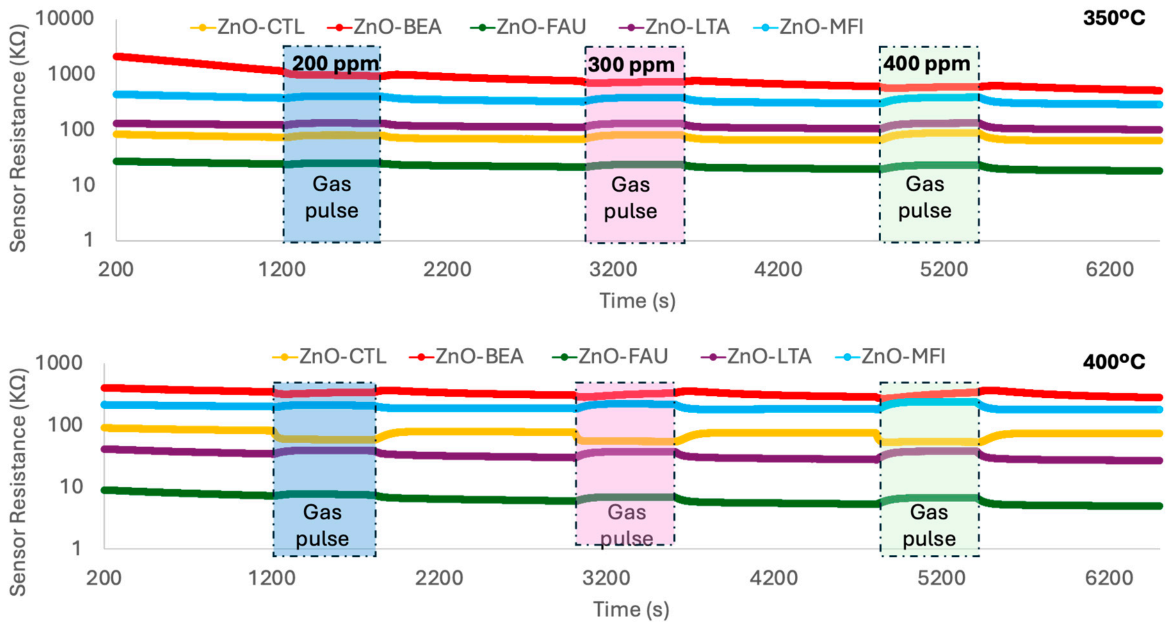


**Figure S5 – ZnO-based sensor resistance to 200, 300, and 400 ppm carbon monoxide between 300 and 400°C.**

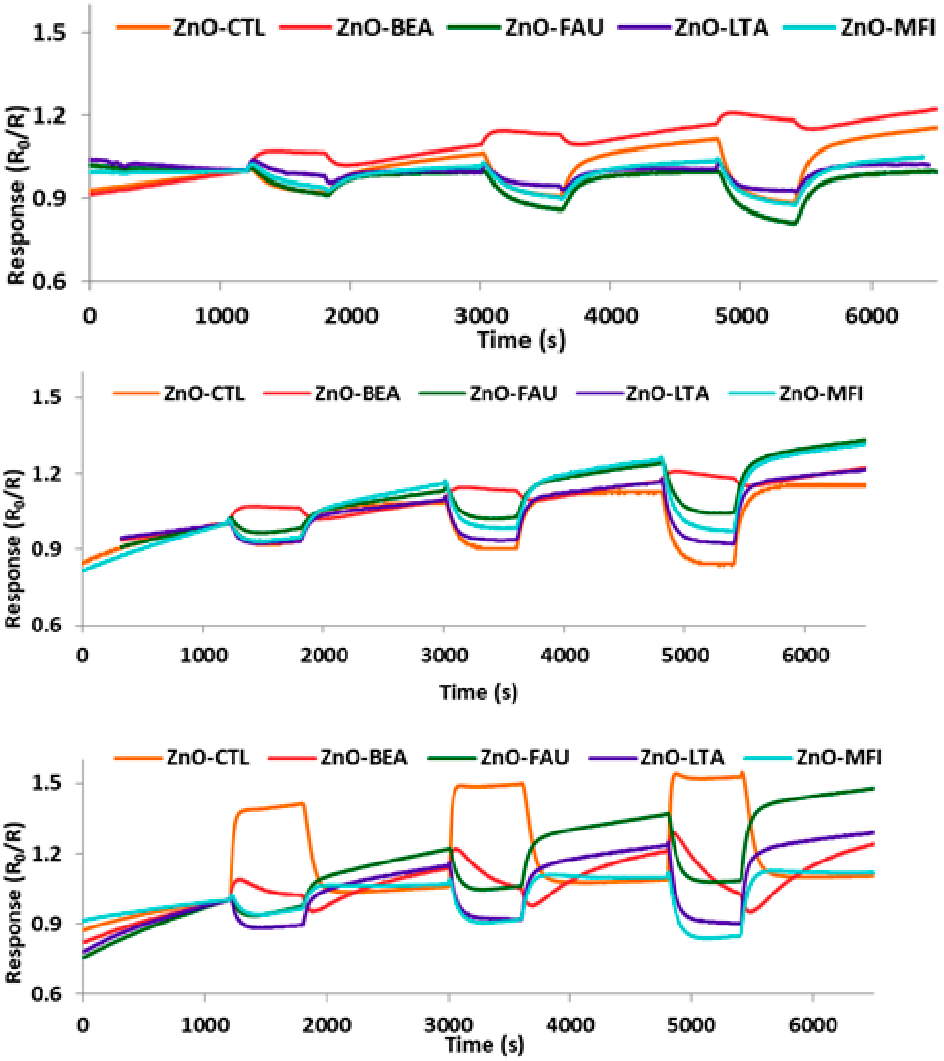


**Figure S6 – ZnO and zeolite-modified sensors' response towards 200, 300, and 400 ppm carbon monoxide between 300 and 400°C.**

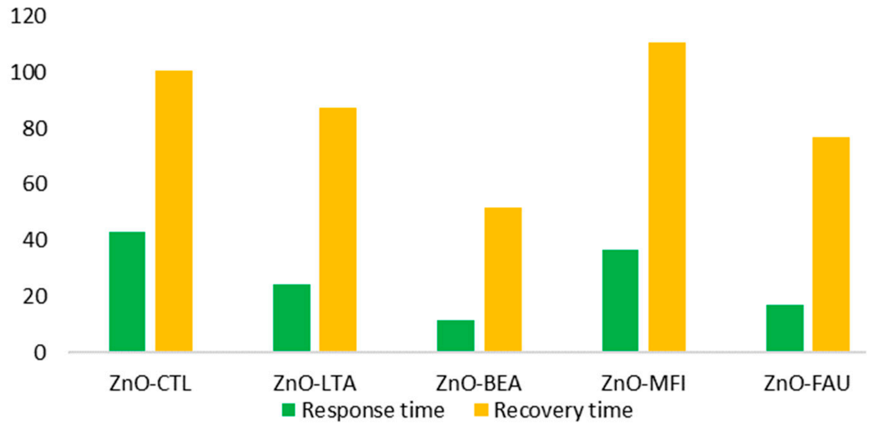


**Figure S7 - Response and recovery times of ZnO sensors towards carbon monoxide gas with different zeolite overlayers at 500 °C. $t_{res}$ refers to the sensor response time (sec) and $t_{rec}$ to the sensor recovery time (s).**

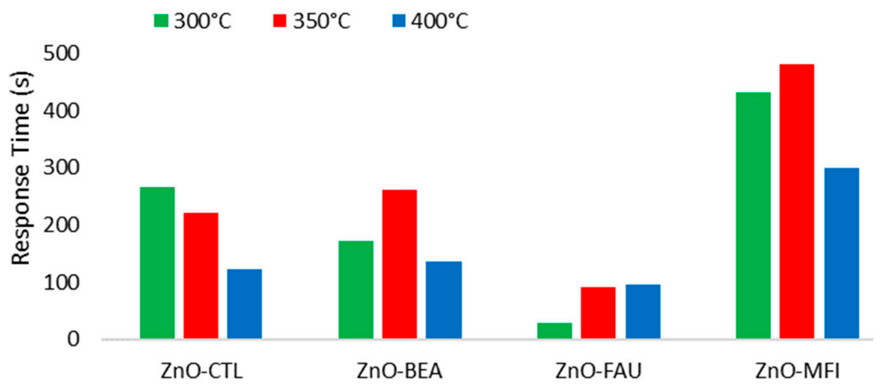


**Figure S8 - The response time of zeolite-modified sensors towards 60 ppm of acetone over a range of temperatures from 300°C to 400°C.**

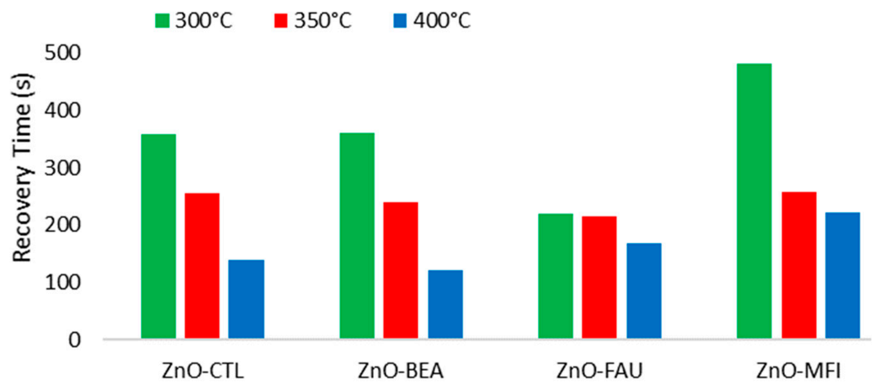


**Figure S9 - The recovery time of zeolite-modified sensors towards 60 ppm of acetone over a range of temperatures from 300°C to 400°C.**

**Table S1 - Comparison of sensor performance in present paper to other published studies on zeolite-modified ZnO.**

| Material | Sensor Fabrication Method | Gas Concentration (ppm) | Temperature (ºC) | Gas Tested | Sensor Response |
|---|---|---|---|---|---|
| **ZnO thin film with zeolite overlayers** [30] | Spin-coating | 0.2 | 97 | Nitrogen Dioxide | 26 |
| **ZnO@ZIF-8 core-shell structure** [29] | Base material: screen-printed \| then ZIF-8 growth on sensor surface using hydrothermal synthesis | 50 | 290 | Acetone | ca. 2 |
| | | 100 | | Ethanol | ca. 2 |
| | | 1000 | | Hydrogen | ca. 5.7 |
| **Zeolite A coated Zn1−XCuXO MOS sensors** [28] | Copper-doped ZnO nanostructures grown using successive ionic layer adsorption and reaction then zeolite A coated on the base sensor using hydrothermal secondary growth method | 0.02 | 55 | Nitric Oxide | ca. 25% |
| **Zeolite thick film overlayers (H-ZSM-5, Na-A, H-Beta, H-Y) on ZnO – This Paper** | Screen-printed zeolite Beta as overlayer of ZnO base material | 40 | 350 | Ethanol | ca. 8 |
| | Screen-printed zeolite H-Y as overlayer of ZnO base material | 40 | 350 | Ethanol | ca. 3 |
| | Screen-printed zeolite Na-A as overlayer of ZnO base material | 40 | 350 | Ethanol | ca. 60 |
| | Screen-printed zeolite H-ZSM-5 as overlayer of ZnO base material | 40 | 350 | Ethanol | ca. 24 |
| | Screen-printed zeolite Beta as overlayer of ZnO base material | 10 | 350 | Acetone | 18.8 |
| | Screen-printed zeolite H-Y as overlayer of ZnO base material | 10 | 350 | Acetone | 6 |
| | Screen-printed zeolite H-ZSM-5 as overlayer of ZnO base material | 10 | 350 | Acetone | 14 |
| | Screen-printed zeolite Beta as overlayer of ZnO base material | 10 | 400 | Acetone | ca. 50 |
| | Screen-printed zeolite H-Y as overlayer of ZnO base material | 10 | 400 | Acetone | ca. 5 |
| | Screen-printed zeolite H-ZSM-5 as overlayer of ZnO base material | 10 | 400 | Acetone | ca.17 |
| | Screen-printed zeolite Beta as overlayer of ZnO base material | 0.9 | 350 | Nitrogen Dioxide | 3.7 |
| | Screen-printed zeolite H-Y as overlayer of ZnO base material | 0.9 | 350 | Nitrogen Dioxide | 18.1 |
| | Screen-printed zeolite Na-A as overlayer of ZnO base material | 0.9 | 350 | Nitrogen Dioxide | 28.7 |
| | Screen-printed zeolite H-ZSM-5 as overlayer of ZnO base material | 0.9 | 350 | Nitrogen Dioxide | 11.6 |
